# Resolving Structural Variability in Network Models and the Brain

**DOI:** 10.1371/journal.pcbi.1003491

**Published:** 2014-03-27

**Authors:** Florian Klimm, Danielle S. Bassett, Jean M. Carlson, Peter J. Mucha

**Affiliations:** 1 Department of Physics, University of California, Santa Barbara, Santa Barbara, California, United States of America; 2 Institut für Physik, Humboldt-Universität zu Berlin, Berlin, Germany; 3 Department of Mathematics, University of North Carolina, Chapel Hill, Chapel Hill, North Carolina, United States of America; 4 Sage Center for the Study of the Mind, University of California, Santa Barbara, Santa Barbara, California, United States of America; 5 Department of Bioengineering, University of Pennsylvania, Philadelphia, Pennsylvannia, United States of America; 6 Department of Applied Physical Sciences, University of North Carolina, Chapel Hill, Chapel Hill, North Carolina, United States of America; Hamburg University, Germany

## Abstract

Large-scale white matter pathways crisscrossing the cortex create a complex pattern of connectivity that underlies human cognitive function. Generative mechanisms for this architecture have been difficult to identify in part because little is known in general about mechanistic drivers of structured networks. Here we contrast network properties derived from diffusion spectrum imaging data of the human brain with 13 synthetic network models chosen to probe the roles of physical network embedding and temporal network growth. We characterize both the empirical and synthetic networks using familiar graph metrics, but presented here in a more complete statistical form, as scatter plots and distributions, to reveal the full range of variability of each measure across scales in the network. We focus specifically on the degree distribution, degree assortativity, hierarchy, topological Rentian scaling, and topological fractal scaling—in addition to several summary statistics, including the mean clustering coefficient, the shortest path-length, and the network diameter. The models are investigated in a progressive, branching sequence, aimed at capturing different elements thought to be important in the brain, and range from simple random and regular networks, to models that incorporate specific growth rules and constraints. We find that synthetic models that constrain the network nodes to be physically embedded in anatomical brain regions tend to produce distributions that are most similar to the corresponding measurements for the brain. We also find that network models hardcoded to display one network property (e.g., assortativity) do not in general simultaneously display a second (e.g., hierarchy). This relative independence of network properties suggests that multiple neurobiological mechanisms might be at play in the development of human brain network architecture. Together, the network models that we develop and employ provide a potentially useful starting point for the statistical inference of brain network structure from neuroimaging data.

## Author Summary

White matter tracts crisscrossing the human cortex are linked in a complex pattern that constrains human thought and behavior. Why the human brain displays the complex pattern that it does is a fascinating open question. Progress in uncovering generative mechanisms for this architecture requires greater knowledge about mechanistic drivers of anatomical networks. Here we contrast network properties derived from images of the human brain with 13 synthetic network models investigated in a progressive, branching sequence, chosen to probe the roles of physical embedding and temporal growth. We characterize both the empirical and synthetic networks using network diagnostics presented here in statistical form, as scatter plots and distributions, to reveal the full range of variability of each measure. We find that synthetic models that constrain the network nodes to be physically embedded in anatomical brain regions tend to produce distributions that are most similar to the corresponding measurements for the brain. We also find that network models hardcoded to display one network property do not in general simultaneously display a second, suggesting that multiple neurobiological mechanisms drive human brain network development. The network models that we develop and employ enable statistical inference of brain network structure from neuroimaging data.

## Introduction

Increasing resolution of noninvasive neuroimaging methods for quantifying structural brain organization in humans has inspired a great deal of theoretical activity [1]–[4], aimed at developing methods to understand, diagnose, and predict aspects of human development and behavior based on underlying organizational principles deduced from these measurements [5]–[7]. Ultimately, the brain is a network, composed of neuronal cell bodies residing in cortical grey matter regions, joined by axons, protected by myelin. Diffusion-weighted magnetic resonance imaging methods trace these white matter connections, based on the diffusion of water molecules through the axonal fiber bundles. While resolution has not reached the level of individual neurons and axons, these methods lead to reliable estimates of the density of connections between regions and fiber path lengths. The result is a weighted adjacency matrix, with a size and complexity that increases with the resolution of the measurements [8], [9].

The immense complexity of this data makes it difficult to directly deduce the underlying mechanisms that may lead to fundamental patterns of organization and development in the brain [10]. As a result, comparison studies with synthetic network models, employing quantitative graph statistics to reduce the data to a smaller number of diagnostics, have provided valuable insights [11]–[15]. These models and statistics provide a vehicle to compare neuroimaging data with corresponding measurements for well-characterized network null models. However, the methods are still in development [16]–[18], and vulnerable to the loss of critical information through oversimplification of complex, structured data sets, by restricting comparisons to coarse measurements that ignore variability [10], [19], [20].

Two critical questions motivate development of network methodologies for the brain. The first question focuses on predictive statistics: Are there graph metrics that may ultimately be useful in parsing individual differences and diagnosing diseases? Comparing empirical brain data to benchmark null models can establish the statistical significance of a topological property [21]–[23], and normalizing a topological property by its null model surrogate can be a useful preprocessing step prior to the determination of statistical differences in brain network structure between groups [16]. The second question focuses on network characteristics from a fundamental, development and evolutionary perspective: What organizational principles underlie growth in the human brain? Here comparing empirical brain data to simplified model networks that have been created to capture some aspect of, for example, neurodevelopmental growth rules [24], neuronal functions [11], or physiological constraints [25] may aid in developing a mechanistic understanding of the brain's network architecture (e.g., [26]–[28]). Both efforts require a basic understanding of the topological similarities and differences between synthetic networks and empirical data.

**Figure 1:**
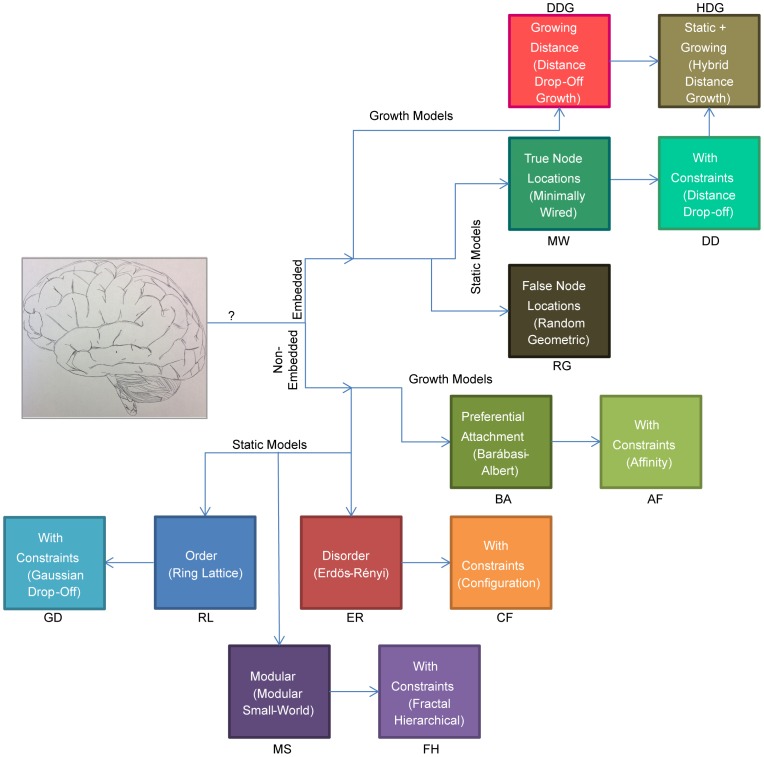
Branching structure of synthetic model examination. We distinguish between synthetic networks that are constructed based on rules for connectivity between nodes (*non-embedded*), and those that constrain nodes to reside in anatomical brain regions (*embedded*). We further distinguish between synthetic networks that are obtained from static ensembles (*static*), and those that are obtained from growth rules (*growing*). In the non-embedded case, we explore common benchmark networks including regular lattice, Erdös-Rényi, and small-world models as well as a second set of networks that are based on these benchmarks but that also employ additional constraints. For growing models, we explore the Barábasi-Albert model and introduce an *affinity* model inspired by preferential attachment-like properties of neuronal growth. In the embedded case, we distinguish between models that utilize true or false node locations (i.e., models derived from a spatial embedding independent of the known, physical node locations) and explore several growing models inspired by hypotheses regarding wiring minimization in brain development [26], [28], [29].

In this paper, we perform a sequence of detailed, topological comparisons between empirical brain networks obtained from diffusion imaging data and 13 synthetic network models (see Table 1). The models are investigated in a tree-like branching order, beginning with the simplest, random or regular graphs, and progressively adding complexity and constraints (see Figure 1). The objective of this investigation is to determine, in a controlled, synthetic setting, the impact of network properties on the topological measurements. Our goal is not to create a definitive network model of the brain, but to gain an intuition for structural drivers of network statistics and to create a battery of null models to be used in statistical comparisons of brain networks.

**Table 1:** Network models names, abbreviations, intuitive descriptions, and associated references.

Model Name	Abbreviation	Description	Citation
**Non-embedded**			
*Static*			
Erdös-Rényi	ER	Uniform connection probability	[60]
Configuration	CF	Random rewiring preserving degree distribution	[110]
Ring Lattice	RL	Fixed degree to *k* nearest neighbors	[62]
Gaussian Drop-Off	GD	Gaussian drop-off in edge density with increasing distance from the diagonal	[60], [111]
Modular Small-World	MS	Fully connected modules linked together by evenly distributed random connections	[60]
Fractal Hierarchical	FH	Modular structure across *n* hierarchical levels; connection density decays as 1/(*E^n^*)	[60]
*Growth*			
Barabási-Albert	BA	Network growth by preferential attachment rule	[78]
Affinity	AF	Two-step preferential attachment growth with hardcoded assortativity and hierarchy	
**Embedded**			
*Static*			
Random Geometric	RG	Wire together random node locations with shortest possible connections	[79]
Minimally Wired	MW	Wire together true node locations with shortest possible connections	[26]–[28]
Distance Drop-Off	DD	Wire together true node locations with a probability that drops off with distance between nodes	[82]
*Growth*			
Distance Drop-Off Growth	DDG	Network growth by distance drop-off rule	
Hybrid Distance Growth	HDG	Minimally wired network that grows with distance drop-off rule	

At the coarsest level in the model hierarchy, we distinguish between synthetic networks that are constructed purely based on rules for connectivity between nodes (non-embedded), and those that constrain nodes to reside in anatomical brain regions (embedded) (see Figure 1). While non-embedded models are frequently used for statistical inference, recent evidence has suggested that physical, embedding constraints may have important implications for the topology of the brain's large-scale anatomical connectivity [2], [8], [22], [26]–[29]. By examining both non-embedded and embedded models, we hope our results will help to guide the use, development, and understanding of more biologically realistic models for both statistical and mechanistic purposes [23], [30].

A second important classification of the synthetic models in our study separates those obtained from static ensembles with fixed statistical properties and those generated using mechanistic growth rules (see Figure 1). While algorithms for generating networks based on static sampling and growth rules ultimately both produce ensembles of fixed graphs for our comparison with data, additional constraints imposed by underlying growth rules may facilitate understanding of mechanisms for development and evolution in the brain as well as other biological and technological networks.

To compare the models with brain data, we employ a particular subset of the many network diagnostics that have been proposed as measures of network topology [31], specifically chosen to highlight the regional variability and multiscale nature of network architecture. Many network diagnostics can be described as *summary diagnostics*, in which a property of the network organization is reduced to a single diagnostic number. Examples include average path length and average clustering coefficient. However, the comparison of summary diagnostics between real and model networks can be difficult to interpret [32] because they often hide the granularity at which biological interpretations can be made. To maximize the potential for a mechanistic understanding, we instead study diagnostics that provide distributions, visualized and analyzed by two-dimensional curves or scatter plots where the regional variability of network structure is readily apparent. The following four diagnostic relationships are obtained from a distribution of values over network nodes or topological scales: hierarchy [33], degree assortativity [34], topological Rentian scaling [35], [36], and the topological fractal dimension [37]. Each of these inherently *relational* properties has previously been investigated in the context of anatomical brain networks in humans [28], [38], [39]. In this paper, we use them to examine the differences between empirically derived anatomical brain networks and synthetic network models.

## Materials and Methods

### Data

**Figure 2:**
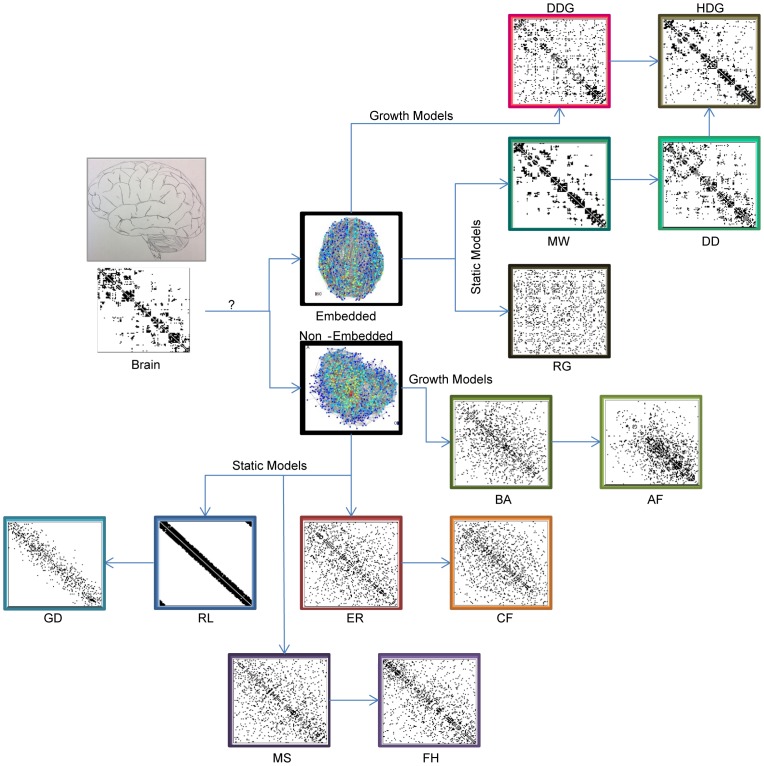
Adjacency matrices for brain and synthetic models. Example adjacency matrices are provided for the brain and for the 13 synthetical network models described in Figure 1. In the empirical brain data and the non-embedded null models, network nodes are ordered along the *x* and *y*-axes to maximize connectivity along the diagonal, as implemented by the *reorderMAT.m* function in the Brain Connectivity Toolbox [60]. In the embedded models, nodes are listed in the same order as they are in the empirical brain data. Abbreviations are as listed in Table 1.

We utilize previously published diffusion spectrum imaging data [39] to examine the network structure of anatomical connectivity between cortical regions in the human brain. In this data, the direct pathways between *N* = 998 cortical regions of interest are estimated using deterministic white matter tractography in 5 healthy human participants [39]. This procedure results in an *N*×*N* weighted undirected adjacency matrix **W** representing the network, with elements *W_ij_* indicating the (normalized) number of streamlines connecting region *i* to region *j* (see Figure 2).

The organization of white matter tracts can be examined at two distinct levels of detail: topological and weighted. Studies of the topological organization of brain anatomy focus on understanding the presence or absence of white matter tracts between regions [26]–[28], while studies of the weighted organization focus on understanding the strength of white matter connectivity between those regions. In this paper, we explore the topological organization of white matter connectivity between cortical regions. In future work we plan to build additional constraints into our models that will enable a comparison of model and empirical weighted networks.

To study topological organization, we construct the binary adjacency matrix **A** in which the element *A_ij_* is equal to 1 if the employed tractography algorithm identifies any tracts (of any strength) linking region *i* with region *j* (i.e., 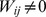). In this data [39], the adjacency matrix **A** is relatively sparse, resulting in a network *density* of 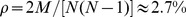, where 
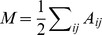
 is the total number of connections present. This estimate of brain network sparsity is consistent with estimates extracted from other similar data sets of comparable network size [8], [40].

Given the potential variability in the topological organization of networks extracted from different individuals [8], [41]–[44], we report results for one individual in the main manuscript and describe the consistency of these results across subjects in the Supplementary Materials.

We also briefly note that while extremely rich, this data set also has its limitations. In particular, the development of high resolution imaging methods and robust tractography algorithms to resolve crossing fibers are fast-evolving areas of research. Novel imaging techniques have for example recently identified the existence of 90-degree turns in white matter tracts [45], a biological marker that we are not sensitive to in our data.

### Network Diagnostics

We measure four network properties including degree assortativity, hierarchy, Rentian scaling, and topological fractal dimension as well as several summary diagnostics, as reported in Table 2.

**Table 2:** Diagnostics of network topology in real and synthetic network models.

Network	mode degree (#)	width of degree distribution min *k_i_*–max *k_i_*	assortativity *r*	hierarchy *β*	global clustering *C* [%]	Rentian scaling *p_T_*	topological fractal dimension *d_B_*	diameter *D*	average shortest path length *P*	modularity	number of communities
***Empirical***											
Brain	**23 & 25 (40)**	**0–87**	**0.149**	**0.247**	**41.5**	**0.745±0.003**	**3.7±0.1**	**6**	**3.1**	0.615	18.46
***Non-embedded***											
*Static*											
ER	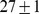 ( 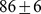 )	( 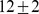 )–( 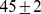 )	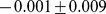	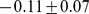	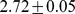	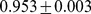	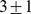	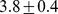	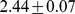	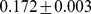	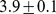
CF	**23 & 25 (40)**	(**0**)**–**(**87**)	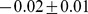	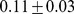	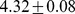	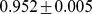	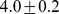	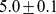	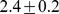	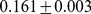	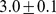
RL	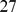 ( 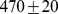 )	( 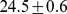 )–( 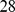 )	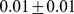	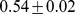	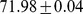	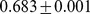	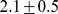	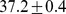	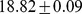	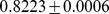	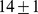
GD	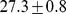 ( 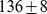 )	( 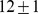 )–( 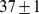 )	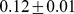	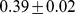	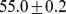	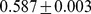	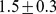	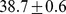	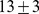	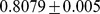	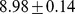
MS	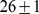 ( 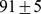 )	( 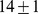 )–( 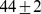 )	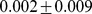	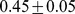	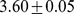	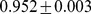	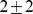	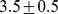	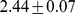	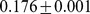	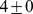
FH	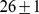 ( 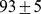 )	( 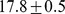 )–( 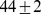 )	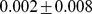	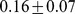	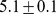	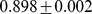	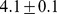	4	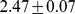	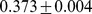	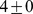
*Growth*											
BA	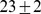 ( 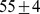 )	( 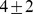 )–( 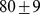 )	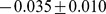	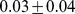	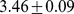	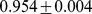	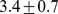	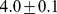	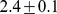	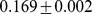	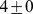
AF	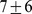 ( 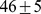 )	( 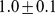 )–( 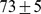 )	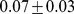	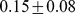	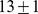	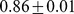	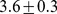	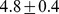	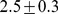	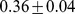	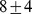
***Embedded***											
*Static*											
RG	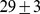 ( 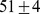 )	( 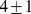 )–( 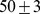 )	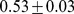	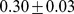	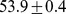	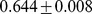	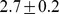	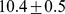	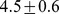	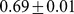	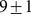
MW	28 (65)	7–49	0.3971	0.3657	54.66	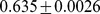	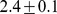	11	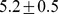	0.7	8
DD	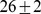 ( 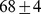 )	( 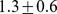 )–( 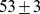 )	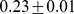	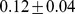	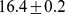	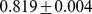	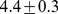	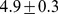	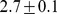	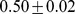	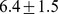
*Growth*											
DDG	**16**±**6** (**40**±**5**)	( 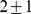 )–( 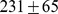 )	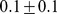	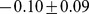	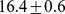	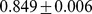	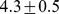	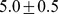	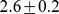	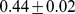	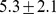
HDG	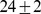 ( 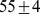 )	( 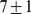 )–( 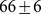 )	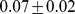	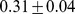	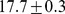	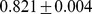	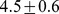	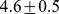	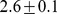	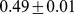	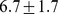

In the second column, we report mode degree and the number of nodes identified with that degree (note that the mean degree is identical for all synthetic and real networks by construction). In the remaining columns, we report the mean and standard deviation of network diagnostics values, calculated over 100 realizations of each synthetic network model. We report the empirical value for the brain data (see Supplementary Material for additional results for other participants). Note that the algorithms for determining fractal dimension and Rentian scaling are not deterministic and therefore multiple estimates can be obtained by applying the algorithms to the same network multiple times. To assess the relative role of different types of variance in the estimated value of diagnostics, we provide Table S2 in the Supplementary Materials, where we report confidence intervals in the fitted values of several diagnostics (fitting variance), standard deviation of the diagnostic value over 100 computations (algorithmic variance), and standard deviation of the diagnostic value over 100 realizations of the network (model variance).

#### Assortativity

The number of edges emanating from node *i* is referred to as its degree, denoted by *k_i_*. The degree assortativity of a network, or more simply ‘assortativity’ here, is defined as the correlation between a node's degree and the mean degrees of that node's neighbors which can be calculated as
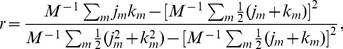
(1)where 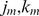 are the degrees of the nodes at either end of the *m^th^* edge, with 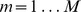 [46]. The assortativity measures the likelihood that a node connects to other nodes of similar degree (leading to an assortative network, 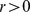) or to other nodes of significantly different degree (leading to a disassortative network, 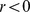). Social networks are commonly found to be assortative while networks such as the internet, World-Wide Web, protein interaction networks, food webs, and the neural network of *C. elegans* are disassortative [34].

#### Hierarchy

The hierarchy of a network is defined quantitatively by a relationship between the node degree and the local clustering coefficient 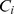 [47]. For each individual node *i*, 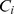 is defined as:
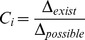
(2)where 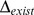 is the number of existing triangle subgraphs that include node *i*, and 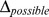 is the number of node triples containing node *i*. Using this local definition, the clustering coefficient of the graph *C* as a whole (a summary diagnostic) is defined as the mean of *C_i_* over all nodes in the network.

The definition of hierarchy is based on a presumed power law relationship between the local clustering coefficient *C_i_* and the degree *k_i_* of all nodes *i* in the network [33]:
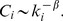
(3)For a given network, the best fit to the scaling exponent *β* is referred to as the network hierarchy.

#### Topological Rentian scaling

In contrast to the physical Rent's rule [35], the topological Rent's rule is defined as the scaling of the number of nodes *n* within a topological partition of a network with the number of connections or edges, *e*, crossing the boundary of that topological partition. If the relationship between these two variables is described by a power law (i.e., 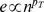), the network is said to show topological Rentian scaling, or a fractal topology, and the exponent of this scaling relationship is known as the topological Rent exponent, 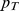 [48]. Thus, higher values of the topological Rent exponent are indicative of a higher dimensional network topology. Pragmatically, to determine 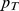, we follow the procedure outlined in [36] where topological partitions are created by a recursive min-cut bi-partitioning algorithm that ignores spatial locations of network nodes [28].

#### Topological fractal dimension

The topological Rent's exponent described above is related to the topological dimension, 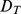, of the network according to the inequality 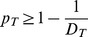 [48]. To directly quantify the topological dimension of a network, we evaluate its topological invariance under length-scale transformations [37]. We employ a box-counting method [49] in which we count the number of boxes 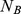 of topological size 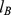 that are necessary to cover the network. The fractal dimension of the network can then be estimated as the exponent 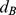 of the putative power law relationship
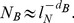
(4)The fractal dimension of a network is a measure of the network's complexity. We note that the process of tiling the network into boxes of different sizes is non-deterministic. To account for this variability, we report mean values of 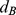 over 50 different tilings of a given network.

#### Additional quantities of interest

In Table 2, we list several summary diagnostics of interest to complement our analysis of relational properties. These include the average path length, the network diameter, the maximum modularity, and the number of communities. The average path length between node *i* and *j* is defined as the shortest number of edges one would have to traverse to move from node *i* to node *j* [50]. The path length of an entire network, *P*, is then defined as the average path length from any node to any other node in the network: 
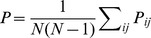
, while the maximal path length between any two pairs of nodes is called the *diameter* 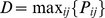.

To determine the maximum modularity and number of communities, we perform community detection by optimizing the modularity quality function [34], [51]–[54]

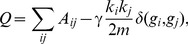
(5)where node *i* is assigned to community *g_i_*, node *j* is assigned to community *g_j_*, the Kronecker delta 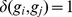 if 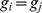 and it equals 0 otherwise, 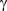 is a resolution parameter (which we set to the common choice of 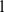, although other values of γ can be used to examine communities at multiple scales [53], [55], [56]), *k_i_* is the degree of node *i*, *m* is the mean degree of the network, and 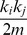 is the expected weight of the edge connecting node *i* to node *j* under the Newman-Girvan null model [51]. We use a Louvain-like [57] algorithm to perform the optimization of *Q* (an optimization which is NP-hard [53], [54], [58]) over different partitions to identify community structure in the network [59]. In Table 2, we report both the maximum modularity and the number of communities present in the partition that maximized *Q*. We note that we performed the maximization of *Q* 100 times and we report the variance in values of *Q* and the number of communities #*_com_* over these 100 optimization in Table S2 in the Supplementary Materials.

### Statistics, Software, and Visualization

All computational and basic statistical operations (such as t-tests and correlations) were implemented using MATLAB (2009b, The MathWorks Inc., Natick, MA) software. Graph diagnostics were estimated using a combination of in-house software, the Brain Connectivity Toolbox [60], and the MATLAB Boost Graph Library (http://www.stanford.edu/~dgleich/programs/). To perform the recursive topological partitioning employed in the examination of topological Rentian scaling, we used the software tool *hMETIS* [61].

Several of the network models that we investigate include one or more tunable parameters affecting the details of the generated graphs. These include the Barabási-Albert, affinity, and hybrid distance growth models. To compare these network models to the data, we optimized parameter values to minimize the difference between the model network and the empirical brain network. Specifically, we used the Nelder-Mead simplex method, which is a derivative-free optimization method, that minimizes the value of a difference metric 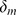 between the two networks. We chose to let 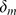 be the sum of the absolute relative difference of nine of the network characteristics reported in Table 2 (clustering coefficient *C*, path length *P*, diameter *D*, degree assortativity *r*, hierarchical parameter *β*, topological Rentian exponent *p_T_*, topological fractal dimension *d_B_*, modularity *Q*, and number of communities #*_com_*). Alternative choices for the difference metric could weight some network characteristics to a greater or lesser degree than others. However, because we do not *a priori* have a rubric by which to determine the biological relevance of a single network diagnostic in comparison to others, we chose not to utilize such a weighting scheme.

## Results

In this section we individually compare topological network diagnostics calculated for the empirical brain data to each of the 13 network models that appear in Figures 1 and 2. We proceed through the catalog of synthetic models along the branches illustrated in Figure 1. We begin with the simplest models (i.e. non-embedded, static, random and regular), and incrementally add structure, constraints, growth mechanisms, and embedding in order to isolate how these additional features impact the measured diagnostics.

**Figure 3:**
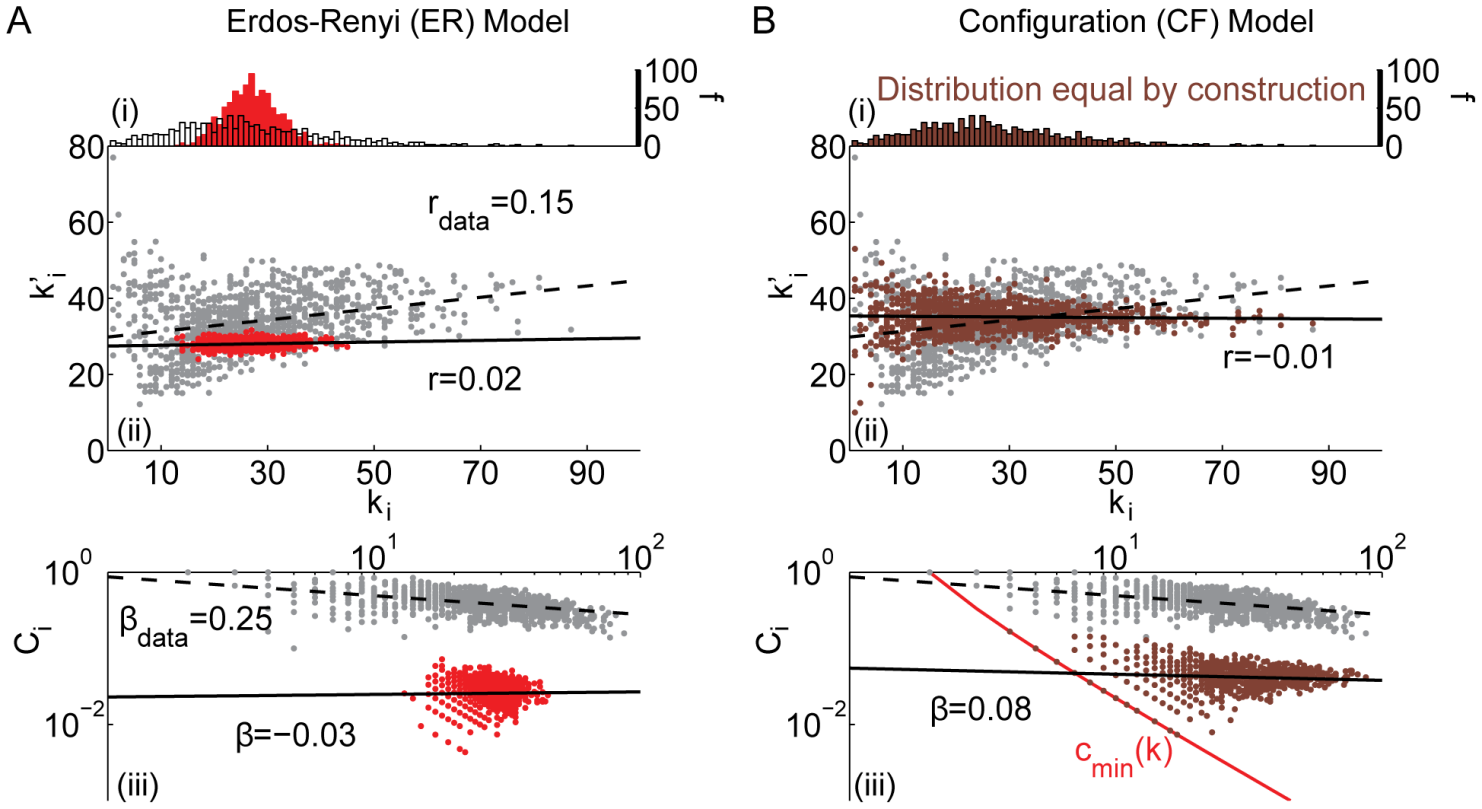
Comparison between the *(i)* degree distribution (number *f* of nodes with a given degree *k_i_*), *(ii)* assortativity (correlation between a node's degree *k_i_* and the mean degree of that node's neighbors 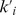, summarized by parameter *r*), and *(iii)* hierarchy (the relationship between the clustering coefficient *C_i_* and the degree *k_i_* over all nodes in the network, summarized by parameter *β*) of the *(A)* Erdös-Rényi and *(B)* configuration model with conserved degree distribution models and the same diagnostics of the brain anatomical data (grey). Black lines indicate best linear fit to the data (dashed) and model (solid) networks. In panel *(B)* the lower (nonzero) bound on the clustering coefficient—which corresponds to the presence of only one triangle—as a function of degree is indicated by the red line.

**Figure 4:**
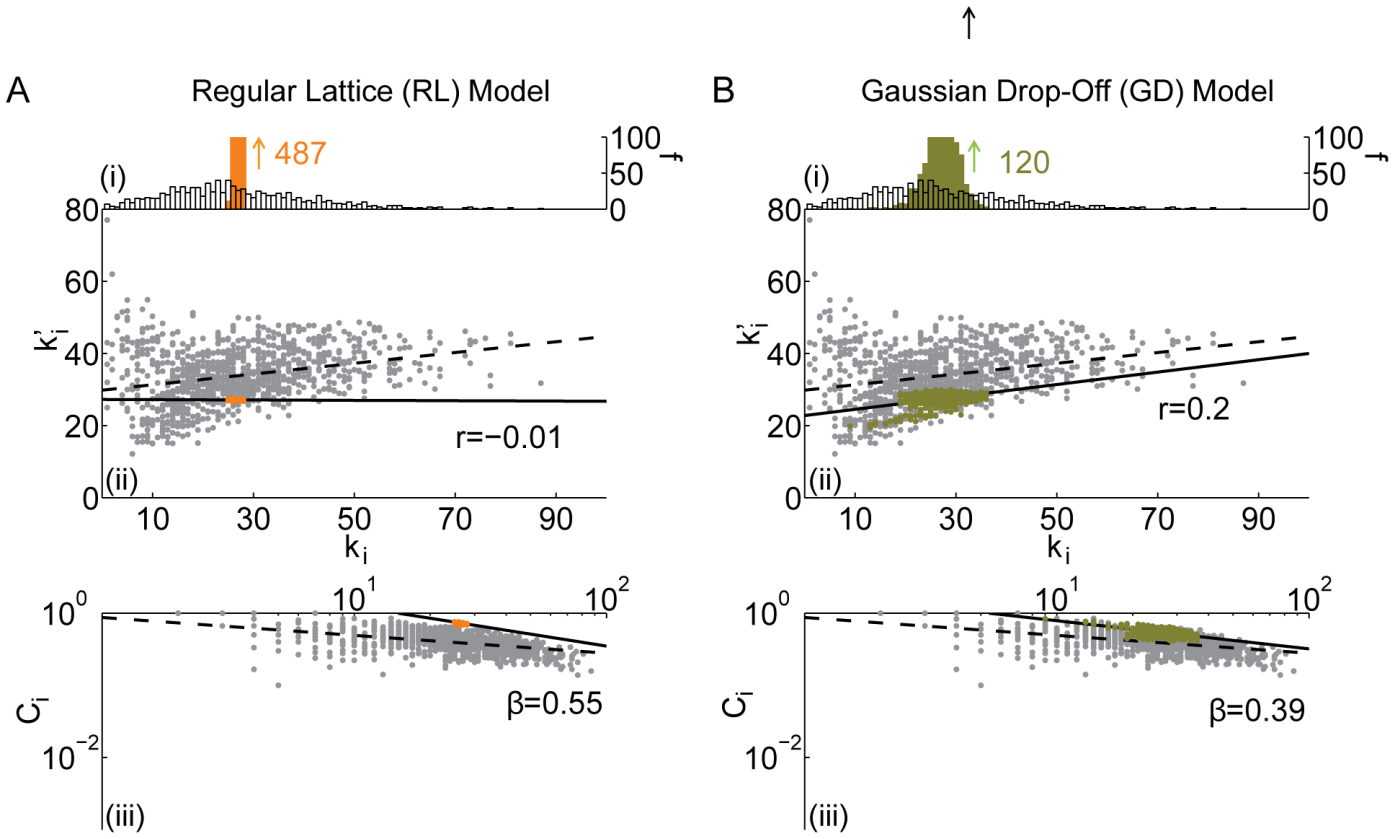
Comparison between the *(i)* degree distribution (number *f* of nodes with a given degree *k_i_*), *(ii)* assortativity (correlation between a node's degree *k_i_* and the mean degree of that node's neighbors 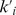, summarized by parameter *r*), and *(iii)* hierarchy (the relationship between the clustering coefficient *C_i_* and the degree *k_i_* over all nodes in the network, summarized by parameter *β*) of the *(A)* ring lattice and *(B)* Gaussian drop-off models and the same diagnostics in the brain anatomical data (grey). Black lines indicate best linear fit to the data (dashed) and model (solid) networks.

**Figure 5:**
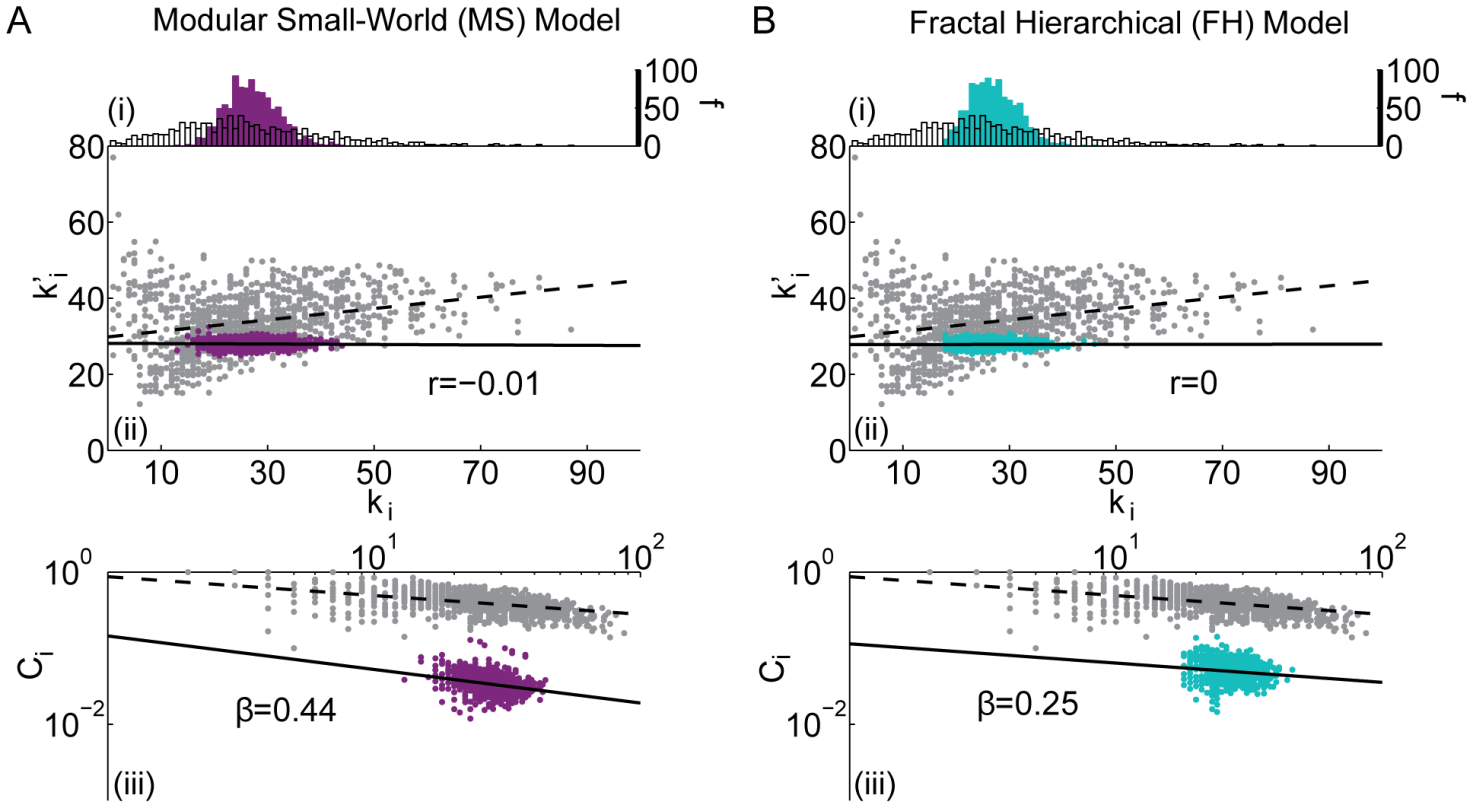
Comparison between the *(i)* degree distribution (number *f* of nodes with a given degree *k_i_*), *(ii)* assortativity (correlation between a node's degree *k_i_* and the mean degree of that node's neighbors 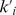, summarized by parameter *r*), and *(iii)* hierarchy (the relationship between the clustering coefficient *C_i_* and the degree *k_i_* over all nodes in the network, summarized by parameter *β*) of the *(A)* modular small-world and the *(B)* fractal hierarchical models and the same diagnostics in the brain anatomical data (grey). Black lines indicate best linear fit to the data (dashed) and model (solid) networks.

**Figure 6:**
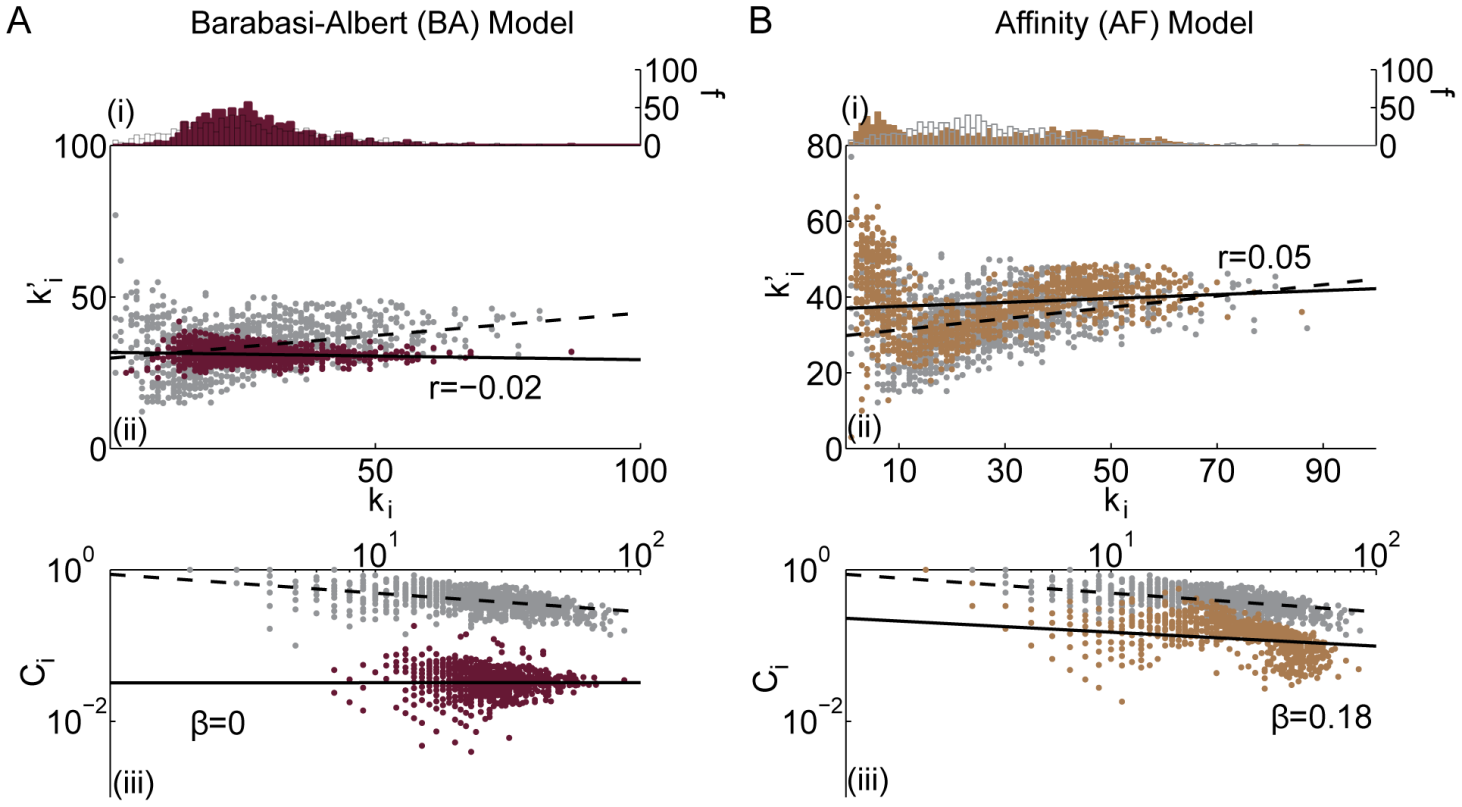
Comparison between the *(i)* degree distribution (number *f* of nodes with a given degree *k_i_*), *(ii)* assortativity (correlation between a node's degree *k_i_* and the mean degree of that node's neighbors 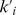, summarized by parameter *r*), and *(iii)* hierarchy (the relationship between the clustering coefficient *C_i_* and the degree *k_i_* over all nodes in the network, summarized by parameter *β*) of the *(A)* Barabási-Albert and *(B)* affinity models and the same diagnostics in the brain anatomical data (grey). Black lines indicate best linear fit to the data (dashed) and model (solid) networks. In panel *(B)*, the parameter values used for the affinity model are the following: 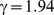, 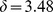, and 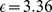.

**Figure 7:**
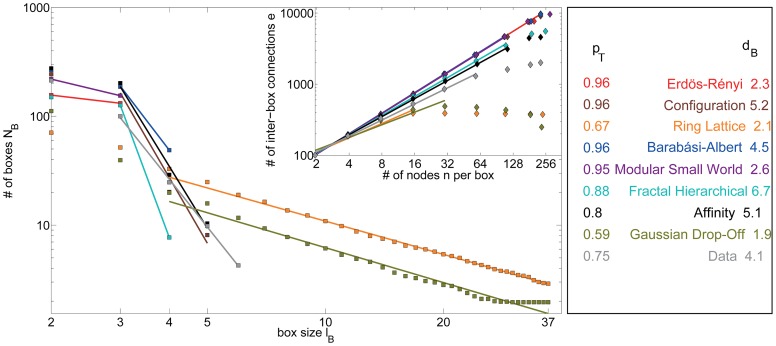
Diagnostics estimating the topological dimension. *(Main Panel)* The number of boxes as a function of the topological size of the box, as estimated using the box-counting method [49] (see the Materials and Methods section) for the real and synthetic networks. *(Inset)* The topological Rentian scaling relationship between the number of edges crossing the boundary of a topological box and the number of nodes inside of the box (see the Materials and Methods section) for the real and synthetic networks. Lines indicate data points included in fits reported in Table 2.

**Figure 8:**
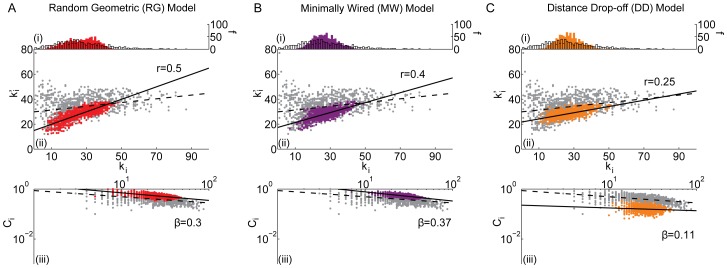
Comparison between the *(i)* degree distribution (number *f* of nodes with a given degree *k_i_*), *(ii)* assortativity (correlation between a node's degree *k_i_* and the mean degree of that node's neighbors 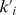, summarized by parameter *r*), and *(iii)* hierarchy (the relationship between the clustering coefficient *C_i_* and the degree *k_i_* over all nodes in the network, summarized by parameter *β*) of the *(A)* random geometric (RG), *(B)* minimally wired (MW), and *(C)* distance drop-off (DD) models and the same diagnostics in the brain anatomical data (grey). Black lines indicate best linear fit to the data (dashed) and model (solid) networks.

**Figure 9:**
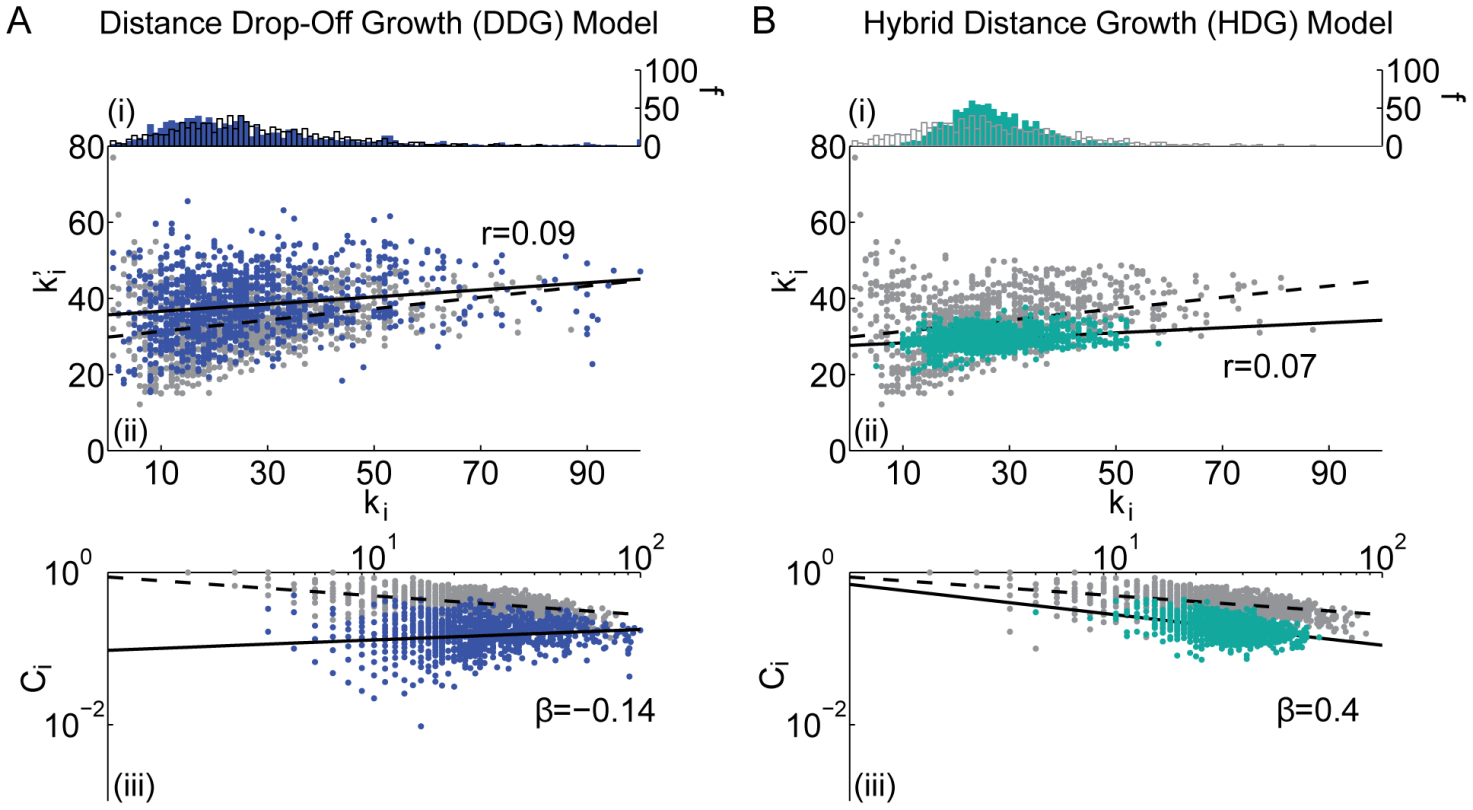
Comparison between the *(i)* degree distribution (number *f* of nodes with a given degree *k_i_*), *(ii)* assortativity (correlation between a node's degree *k_i_* and the mean degree of that node's neighbors 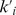, summarized by parameter *r*), and *(iii)* hierarchy (the relationship between the clustering coefficient *C_i_* and the degree *k_i_* over all nodes in the network, summarized by parameter *β*) of the *(A)* distance drop-off growth (DDG) and the *(B)* hybrid distance growth (HDG) models and the same diagnostics in the brain anatomical data (grey). Black lines indicate best linear fit to the data (dashed) and model (solid) networks. In panel *(B)*, we use 4000 minimized wired seed edges.

**Figure 10:**
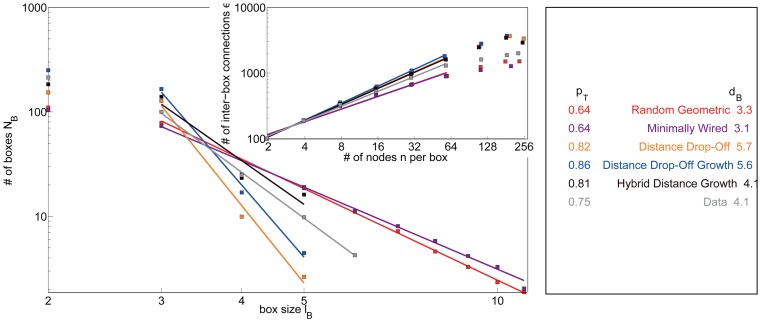
Diagnostics estimating the topological dimension. *(Main Panel)* The number of boxes as a function of the topological size of the box, estimated using the box-counting method [49] (see the Materials and Methods section) for the real and embedded model networks. *(Inset)* The topological Rentian scaling relationship between the number of edges crossing the boundary of a topological box and the number of nodes inside of the box (see the Materials and Methods section) for the real and embedded model networks. Lines indicate data points included in fits reported in Table 2.

For each network we present statistical results for three diagnostics (see Materials and Methods Section): (i) the degree distribution 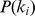 vs. 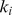, (ii) the mean node degree of the neighboring nodes vs. node degree 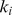 for each node *i* (used to calculate assortativity), and (iii) the local clustering coefficient 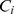 vs. node degree 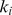 for each *i* (used to calculate hierarchy). In Figures 3–6, the results for the empirical brain network are shown in gray and the corresponding results for each of the synthetic *non-embedded* network models are shown in a contrasting color on the same graph to facilitate comparisons. In addition, we illustrate the scaling relationships used to evaluate Rentian scaling and the topological dimension of each network (see Figure 7). Corresponding results for the synthetic *embedded* network models are provided in Figures 8–9 and 10.

For our comparisons, we group the models first into the set of non-embedded models, followed by the embedded models and we further group results according to the branches of inquiry outlined in Figures 1 and 2. For each model we briefly describe our method for generating the synthetic network, followed by a description of the diagnostics compared to the empirical results.

### Non-embedded Network Models

We begin by comparing the network organization of the brain's anatomical connectivity with that of 8 network models whose structure is not *a priori* constrained to accommodate a physical embedding of the nodes in cortical areas. (In the next subsection, we will examine 5 embedded network models.) The non-embedded network models include an Erdös-Rényi graph, a configuration model with the same degree distribution as the empirical network, a ring lattice graph, a modular small-world graph, a fractal hierarchical graph, a Gaussian drop-off graph, a Barabási-Albert graph, and an affinity graph (see Figure 2 for associated example adjacency matrices for these graphs and Table 1 for abbreviations of model names). These models range from disordered to ordered (e.g., the Erdös-Rényi and regular lattice models) with a range of mesoscale organization for intermediate cases (e.g., modular small-world and fractal hierarchical models) which influence the network diagnostics, and (dis)similarities to corresponding measurements for the brain.

#### Static non-embedded models

*Erdős-Rényi (ER) model:* The Erdős-Rényi (ER) model is an important benchmark network that is often used as a comparison null model for statistical inference. Specifically, we consider the ‘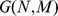 model’ where the ER graph is constructed by connecting pairs chosen uniformly at random from *N* total nodes until *M* edges exist in the graph [62]. The degree distribution generated by this procedure is, as expected, relatively symmetric about the mean degree 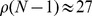 (see Figure 3A(i)).

The ER model is a poor fit to brain anatomical connectivity (see Figure 3A). The degree distribution is much more sharply peaked than the corresponding distribution for the brain. For the ER graph, the variance is approximately equal to the mean degree, while the corresponding data for the brain is more broadly distributed. As a result, the ER network misses structure associated with both high degree hubs and low degree nodes. Because edges are placed at random, organizational properties like assortativity and hierarchy are not observed and—as expected theoretically—the clustering coefficient is smaller and the path length shorter than that of anatomical brain networks (see Table 2).

*Configuration (CF) model*: We next consider a modification of the ER graph that is constrained to have the same degree distribution as the empirical data. We refer to this model as the configuration model (CF). We generate randomized graphs by an algorithm that chooses two existing connections uniformly at random (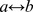 and 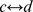) and switches their associations (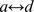 and 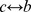) [63].

The CF model agrees with the empirical degree distribution by construction (see Figure 3B(i)). However, it does not fit the higher order association of a node's degree with that node's mean neighbor degree (assortativity) (see Figure 3B(ii)). The average clustering coefficient remains small, although it is larger than that observed in the ER network. In Figure 3B(iii), we observe a small association between the clustering coefficient and degree (hierarchy) which appears to be driven by nodes of small degree. To interpret this finding, we note that the nonzero minimum of the clustering coefficient of a node of degree *k* is given by
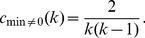
(6)Thus, nodes of small degree tend to have a higher minimum non-zero clustering than nodes of high degree. In comparison to the ER model, the existence of small degree nodes in the CF model leads to an increased diameter of the graph whereas the existence of high degree nodes leads to the maintenance of a short average path length.

*Ring Lattice (RL) model:* In contrast to the two previous models, the ring lattice (RL) model has a highly ordered topology where each node is connected to its 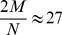 nearest neighbors.

By construction, the degree distribution for the ring lattice is extremely sharply peaked. If the number of edges *M* is divisible by the number of nodes *N*, then all nodes have equal degree, otherwise the remainder is distributed uniformly at random throughout the network, resulting in a very narrow spread in the distribution. The clustering coefficient of the RL model is close to unity, indicating that most neighbors of a node are also connected to each other. The restriction to local connectivity results in a large diameter and long average path length. The small variation in degree induced by the random distribution of the remaining edges is insufficient to induce assortativity (see Figure 4A). Interestingly, however, the RL model displays topological network hierarchy because nodes that have been assigned those remaining edges have a higher than average degree which directly decreases the clustering coefficient of those nodes. It is important to note that the topological properties we observe here are consequences of, rather than artifacts of, the random links that we have distributed through the model. Indeed, the topological role of randomly placed links in networks has been the topic of much recent research (e.g., [47]). In empirically measured networks, it is possible that some randomly distributed links could be either real or spurious [64], [65], and some methods exist to identify and prune spurious links in several real systems [65], [66].

*Gaussian Drop-Off (GD) model:* Compared to the brain, the random and randomized models exhibit lower clustering, and the regular ring lattice exhibits higher clustering. An intermediate topology between these two extremes is obtained by generalizing the concept of local connections from the ring lattice to a stochastically generated network where the density of connections drops off at rate *κ* with increasing distance from the main diagonal of the adjacency matrix.

We chose a value for *κ* by examining the empirical brain data as follows. First, we reordered the adjacency matrix such that the connections (represented by nonzero matrix elements) are predominantly located near the matrix diagonal, using the code reorderMAT.m in the Brain Connectivity Toolbox [60]. We then fit a Gaussian function to the empirical drop-off of the first 400 off-diagonal rows of the reordered brain adjacency matrix [60]. The fit provided an *R* ^2^ value of approximately 0.75.

The very localized structure in this GD model, similar to that observed in an RL model, is softened by the presence of a few long-range connections which decreases the path length and brings the average clustering coefficient closer to that of the data (see Figure 4B). The non-periodic boundary conditions lead to a small subpopulation of nodes with low degree. Because these nodes are neighbors in the adjacency matrix, they tend to be connected to one another, leading to an assortative topology. The same explanation underlies the existence of a hierarchical topology in this GD model, because these low degree boundary nodes predominantly connect with one another.

*Modular Small-World (MS) model:* Small world networks have received a great deal of attention [47] as a conceptual characterization of structure that combines local order with long range connections. While the small world concept is sufficiently general that most networks that are not strictly regular or random fall into this category, small world organization represents more biologically relevant organization than the previous four cases [8], [28], [67], [68]. In addition to the small-world feature, biological networks including those extracted from human brain connectome data [8], [69]–[71] also often display community structure where set of nodes (modules) tend to be highly and mutually interconnected with one another combined with some long-distance connections.

For this study, we construct a synthetic small world network that consists of small, fully-connected modules. While networks composed of large modules could also be studied, we instead chose to use 4-node modules that produced networks displaying large regional heterogeneity in combination with small network building blocks, a pattern consistent with the hierarchical structure observed in brain networks [8], [69], [72]–[74]. The modules in this MS model are randomly linked with one another with enough edges to match the density of the empirical network. This topology leads to high clustering, short path length, and small diameter [60]. The randomly distributed inter-module links emanating from relatively high degree nodes decrease the clustering coefficient of these nodes because nodes in two different modules are unlikely to be otherwise linked. This structure therefore leads to a hierarchical topology (see Figure 5A(iii)). However, because the inter-module links are randomly distributed, nodes that contain such links are no more likely to share an edge with another such node than they are to share a link with any other node in the network. The MS model therefore does not display any observable assortativity (see Figure 5A(ii)).

*Fractal Hierarchical (FH) model:* Like small world networks, fractal hierarchical topology has become a popular classification of networks and applies broadly, at least to some extent, to topologies that are neither regular nor random. Fractal hierarchical structure has been linked to some observed network structure in the brain [8], [69], [72]–[74] and its use in neural network models produces several behaviors reminiscent of empirical neurobiological phenomena [11], [75], [76].

To construct a fractal hierarchical model [33], we follow the approach outlined in [77]. We begin with a set of 4-node modules. We connect pairs of these 4-node modules with a probability 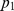 to form 8-node modules. We connect pairs of 8-node modules with a probability 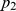 to form 16-node modules. Importantly, the probability *p* of inter-module connections decreases at each level at a prescribed drop-off rate; that is, 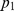 is larger than 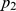, 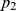 is larger than 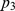, etc. The probabilities at each level are related to one another by a probability drop-off rate. This module-pairing process is repeated until we have formed a 1024-node fractal hierarchical network. To obtain a 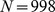 network comparable to the empirical brain data, we chose 26 nodes uniformly at random to delete from the network. If the network contained more (fewer) edges than the empirical network, we repeated the process with an increased (decreased) probability drop-off rate. The algorithm terminates when we obtain an FH model network with the correct number of edges.

The fractal hierarchal network yields extremely similar results to the small world network in terms of the degree distribution, assortativity, and hierarchy (compare Figure 5A with Figure 5B). The striking similarities are surprising given the differences in how the two networks are constructed. While the networks share strong 4-node module building blocks, they differ in their coarser structure. The similarity in the results depicted in Figure 5 suggest that the level-dependent structure in the FH model is not well-captured by these graph properties. Other types of network properties that specifically test for multiresolution phenomenon in brain structure might more readily distinguish between these two synthetic models [56].

#### Growing non-embedded models

In this section we explore two non-embedded growth models (see Figure 1). The first is the Barabási-Albert preferential attachment model and the second is an affinity model which we design to capture assortative and hierarchical structure.

*Barabási-Albert (BA) model:* All models described thus far, with the exception of the configuration model, share a common and critical short-coming: the degree distribution is much narrower than that of the empirical networks. A model that produces a broader distribution of node degrees is the Barabási-Albert model of preferential attachment [78].

To construct a BA network, we begin with a single edge connecting two nodes. Then we iteratively add a single node to the network by linking the new node to *m* existing nodes. The probability of linking the new node to an existing node is given by a preferential attachment function 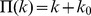 with dimensionless parameter 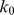 tuning the rate of decrease in the degree distribution. Note that as 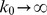, the resultant graph becomes increasingly similar to an ER graph.

To identify a BA model network in this family that best fits the empirical data, we tune 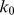 to minimize the difference between the model topology and the empirical topology as described in the Materials and Methods Section. We find that networks constructed using 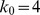 provide the best available fit to the empirical data. The number of edges *m* added with each new node is determined by the total number of edges *M*. This procedure produces networks with low clustering and broad degree distributions, although the number of low-degree nodes is underestimated in comparison to the empirical data (see Figure 6A(i)). Despite the broad degree distribution, the network does not display an assortative or hierarchical topology (see Figure 6A(ii)–(iii)).

*Affinity (AF) model:* We introduce an extension of the BA model that includes constraints specifically designed to capture assortative and hierarchical structure. We define the affinity model by a two step preferential attachment function that does not depend on a node's current degree but instead depends on a dimensionless *affinity* parameter *α*. We begin with *N* nodes, and to each node we assign a unique affinity 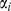 distributed uniformly at random in the interval [0,1]. The value of 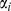 remains unchanged throughout the growth process (see Algorithm 1). We choose a node with probability 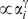 and link that node preferentially to another node *j* with a similar affinity 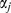. This assortative mixing for affinity ensures degree assortativity. In addition, we choose a preferential attachment function (see Algorithm 1, line 6) such that nodes with small values of affinity (e.g. small degree) are relatively more likely to gain edges with neighbors of similar affinity (and therefore degree) than nodes with large values of affinity. Small degree nodes therefore are more clustered than their high degree counterparts, leading to a hierarchical network structure.

**Algorithm 1.** Growth algorithm for the affinity model.

**Input** :number of nodes *N*

    number of edges *M*

    number of seed edges *M*_0_

    attachment regulators γ, *δ* and 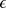

**Output** :Adjacency matrix *A*

1 initialize graph with *N* nodes;

2 connect *M*_0_ pairs of nodes chosen uniformly at random;

3 assign each node an affinity given by 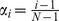;

4 **while** 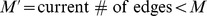 **do**

5 | out of the set of nodes with 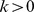, choose a node *i* with probability 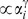

6 | connect node *i* to node *j* (chosen at uniformly at random) with probability

 | 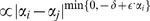

7 **end**

To compare this model to the empirical data, we use a derivative-free optimization method to identify the parameter values for 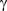, 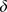, and 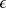 that minimize the difference between the empirical and model networks; see the Materials and Methods Section. The AF model has a very broad degree distribution with a concentration of low degree nodes and an extremely heavy tail of high degree nodes (see Figure 6B(i)). The network is both assortative and hierarchical although the average clustering is lower than that found in the empirical data (see Figure 6B(ii)–(iii)). The randomly chosen edges connecting nodes of high degree induce a small diameter and short path length.

It is not surprising that the AF model provides a better fit for the empirical data for these specific diagnostics than other synthetic networks we have considered so far, since it was specifically constructed to do so. This is, however, no guarantee that this algorithm will capture other network properties of the empirical data. Indeed, the fact that the affinity model also shows a similar topological dimension to the empirical brain network is surprising and interesting (see next section).

#### Diagnostics estimating the topological dimension

In this section, we compare topological measures of the empirical data with the set of 8 non-embedded synthetic networks: 6 static models and 2 growth models.

Using a box-counting method, we estimate the fractal dimension of the empirical and synthetic model networks (see the Materials and Methods Section) and observe three distinct classes of graphs (see Figure 7, main panel). The first group, which includes the Erdös-Rényi and modular small-world models, has a diameter that is too small to allow an adequate estimation of the fractal dimension of the network using the box-counting method. The second group, which includes the Gaussian drop-off and ring lattice models, has a large diameter leading to a small fractal dimension. The third group, which includes the remainder of the models, has a similar diameter to the empirical network and therefore similar fractal dimension. By these comparisons, the affinity model is the best fit to the data and the configuration model is the second best fit.

The Gaussian drop-off and ring lattice models also show distinct topological Rentian scaling in comparison to the other models (see Figure 7, inset). Above a topological box size of 16 nodes, the number of inter-box connections does not increase because the edges are highly localized topologically. All other models display a swifter scaling of the number of edges with the number of nodes in a topological box in comparison to the empirical data. The affinity model displays the most similar scaling to that observed in the empirical data.

### Embedded Network Models

The non-embedded models described in the previous section necessarily ignore a fundamental property of the brain: its embedding in physical space. Spatial constraints likely play an important role in determining the topological properties of brain graphs [22], [26]–[29]. In this section, we explore the topological properties of spatially *embedded graphs* in which the probability of connecting any two nodes in the network depends on the Euclidean distance between them [79]. We explore the same topological diagnostics as we did in the previous section: degree distribution, assortativity, hierarchy, and diagnostics estimating the topological dimension of the network. As a whole, we find that spatially embedded models capture more topological features of the empirical networks than models that lack the physical embedding constraint.

To clarify the distinction between embedded and non-embedded network models, it is necessary to highlight the differences between topological and physical notions of space. Many topological models (such as the Barabási-Albert model) are often described in ways that utilize notions of “local” connections. However, this concept of locality is present in a purely topological sense and not in a geographical sense. Topological models such as the Barabási-Albert model are not derived from spatial embeddings in 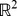 or 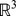 and therefore the nodes of these networks do not have spatial positions and the edges of these networks do not have physical lengths. The nonequivalence of topological and geographic structure is illustrated by the fact that a network topology (e.g., BA) can either remain non-embedded or can be embedded into Euclidean space (e.g., 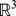) in many different ways: in some embeddings, the topological distance between nodes could correlate with the physical distance between nodes, but in other embeddings one need not observe such a correlation. While the previous section described topological and non-embedded models, in this section we focus on networks that have been embedded into Euclidean space.

#### Static embedded models

*Random Geometric (RG) model:* A random geometric model can be constructed by distributing nodes uniformly at random in a 3-dimensional volume [79]–[81]. We employ a classical neurophysiological embedding in which the x-axis represents the right-left dimension, the y-axis represents the anterior-posterior dimension, and the z-axis represents the superior-inferior dimension. We use a rectangular volume where the length of each side is equal to the maximal Euclidean distance between nodes as measured along that axis and we distribute *N* nodes uniformly at random within this volume. The *M* pairs of nodes with the shortest between-node distance are each connected by an edge.

In the RG model, the heterogeneity of node placement in the volume leads to a broad degree distribution and high clustering between spatially neighboring nodes, leading to a large network diameter and long path length (see Figure 8A(i) and Table 2). Because of the homogeneity of the connection rule, which is identical across all nodes, nodes with high degree (those in close proximity to other nodes) tend to connect to other nodes of high degree and nodes of low degree (those far from other nodes) tend to connect to nodes of low degree, leading to degree assortativity (see Figure 8A(ii)). Nodes at the edges of spatial clusters in the RG model will tend to have high degree but low clustering, leading to a hierarchical topology (see Figure 8A(iii)).

*Minimally Wired (MW) model:* As noted above, nodes in the RG model are placed uniformly at random in a 3-dimensional volume. To add additional anatomical constraints to the model, we can construct a minimally wired model (MW) in which nodes are placed at the center of mass of anatomical brain regions. The *M* pairs of nodes with the shortest between-node distance are then each connected by an edge.

Despite the fact that both models live in 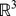, the MW provides an interesting point of comparison to the RG because it allows us to assess what topological properties are driven by the precise spatial locations of brain regions alone. The degree distribution in the MW is narrower than it is in either the RG or the empirical brain network, likely because the brain parcellation used in this study is largely grid-like over the cortex (see Figure 8B(i)). Like the RG, the MW displays degree assortativity and a hierarchical topology (see Figure 8B(ii)–(iii)), and has high clustering and long path length. However, in general the diagnostic relationships extracted from the MW model do not match those of the empirical brain network as well as those extracted from the RG model.

To gain an intuition for the relationships between the observed network statistics in the RG and MW models, it is useful to delineate the similarities and differences between the two models. The RG and MW models are embedded models, meaning that all nodes have a location in physical space, and both models are embedded into 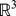. The network topologies that we observe in these models are mathematical consequences of the spatial locations of the nodes combined with the rules for wiring. The RG model contains nodes that are distributed uniformly at random within the brain volume while the MW model contains nodes that are placed at points along the cortical surface (excluding white matter and subcortical structures). Both models stipulate short physical connections but according to different rules. Given the complex combination of similarities and differences between these models, it is not possible to state whether there is a single factor driving the observed differences in network topology without a more in depth study of network models that bridge the topological and geographical space between the RG and MW models.

*Distance Drop-Off (DD) model:* Both the minimally wired and the random geometric models connect only the *M* pairs of nodes with the shortest inter-node distance. These models therefore lack long distance connections which are known to be present in the brain, and have been argued to enable swift communication between distant brain areas [67]. To include this additional biological characteristic, we next study the distance drop-off model (DD) [82], in which we place nodes at empirical brain region locations and then connect pairs of nodes with a probability that depends on the distance *r* between nodes: 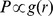. Note that the minimally wired model is a special case of the DD model if we choose 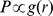 to be a step function with threshold 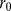. Here, however, we fit a function 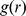 to the connection probability of the empirical data as a function of distance (see Supplementary Material).

The results of the DD model are similar to those that we observed in the case of the minimally wired and random geometric models (see Figure 8C). However, longer distance connections are present in this model which decrease the clustering, path length, diameter, and strength of the assortativity and hierarchy. In general, the diagnostic relationships extracted from the DD model match those of the empirical brain network significantly better than the same diagnostics extracted from the RG and MW models.

#### Embedded growth models

*Distance Drop-Off Growth (DDG) model:* The random geometric, minimally wired, and distance drop-off models all have narrower degree distributions than the empirical data. To expand the degree distribution while still utilizing the empirical node placement and empirically derived probability function 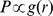, we construct a distance drop-off growth model (DDG). We begin with 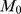 seed edges which we distribute uniformly at random throughout the network. To ensure we have a connected graph, we choose a node *i* uniformly at random from the set of nodes with 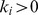. We create an edge between node *i* and node *j*, which is chosen uniformly at random with no constraint on 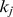, according to the probability 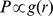. We continue adding edges in this manner until the number of edges in the network is equal to *M*, creating a final DDG model network.

The degree distribution and assortativity of the DDG are surprisingly similar to that observed in the empirical data (see Figure 9A(i)–(ii)). However, the stochasticity of the growth rule induces a decrease in clustering and we do not observe a hierarchical topology (see Figure 9A(iii)). Neither the network diameter nor the path length are significantly altered in comparison to the non-growing distance drop-off model.

*Hybrid Distance Growth (HDG) model:* The minimally wired and distance drop-off growth models display values of summary diagnostics that are most similar to the data (see Table 2). In a final model, we combine facets of both models in a hybrid distance growth model (HDG). We begin by creating a minimally wired model for the 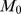 shortest connections. We then use the growing rule of the distance drop-off growth model to add the remaining 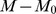 edges to the network. This process can be interpreted as the creation of strongly connected functional modules that afterwards are cross-connected and embedded in the full network. Using a derivative-free optimization method, we estimate that the value of 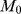 that produces a HDG model network most similar to the empirical network is 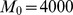; see the Materials and Methods section.

As expected, this HDG model produces a degree distribution, assortativity, and hierarchy in between those produced by the minimally wired and distance drop-off growth models and therefore similar to those observed in the data (see Figure 9B(i)–(iii)). However, the clustering, diameter, and path length remain low in comparison to the empirical data (see Table 2), suggesting that this model does not contain as much local order as the brain.

#### Diagnostics estimating the topological dimension

In this section, we compare topological measures of the empirical data with the set of 5 embedded synthetic networks: 3 static models and 2 growth models.

We observe that the estimates of the topological dimension, using both box-counting and Rentian scaling methods, derived from the physical network models are more similar to the empirical data than those derived from the topological network models (see Figures 7 and 10). The two highly locally clustered networks (the minimally wired and random geometric models) have larger diameters than the brain, decreasing their estimated fractal dimension in comparison. The distance drop-off and distance drop-off growth models are higher dimensional than the empirical data while the hybrid distance growth model displays the same dimension as the empirical data. The hybrid model also produces Rentian scaling with the most similar exponent to that obtained from the empirical data. The identified similarities between models and empirical data are somewhat surprising given that none of these models were explicitly constructed to attain a given topological dimension.

## Discussion

We examined graph diagnostics of 13 synthetic network models and compared them to those extracted from empirically derived brain networks estimated from diffusion imaging data [39]. Some of these models have been defined previously (ER, CF, RL, GD, MS, FH, BA, RG, MW, DD) and others we introduce here for the first time (AF, DDG, HDG). Models which have not previously been applied to the study of diffusion imaging data from the human brain include the RG, DD, AF, DDG, and HDG models. Rather than using solely summary statistics, we characterize distributions and relational properties to more accurately probe the regional variability of network structure. To exercise this more comprehensive analytical approach, we purposefully chose to begin with simple models and iteratively add additional levels of complexity. The inclusion of very simple models (e.g, ER and RL) further enabled us to highlight the structure of the newly defined models (AF, DDG, HDG). In this discussion section, we offer interpretations of many of these models in terms of biologically inspired mechanisms.

We found that in general if a model was hard-coded to display one topological property of the brain (e.g., the degree distribution or the assortativity), it was unlikely to also display a second topological property, suggesting that a single mechanism is unlikely to account for the complexity of real brain network topology. We also observed that those models that employed information about node location and inter-node distances (e.g., embedded network models) were more likely to display similar topological properties to the empirical data than those that were constructed based on topological rules alone (e.g., non-embedded network models). In our examination, three models performed noticeably better than all others: the hybrid distance growing model, the affinity model, and the distance drop-off model. Together, these results provide us with important insights into the relationships between multiple topological network properties. Moreover, these model networks form a catalogue of null tests with a range of biological realism that can be used for statistical inference in static as opposed to dynamic network investigations [23], [70].

**Figure 11:**
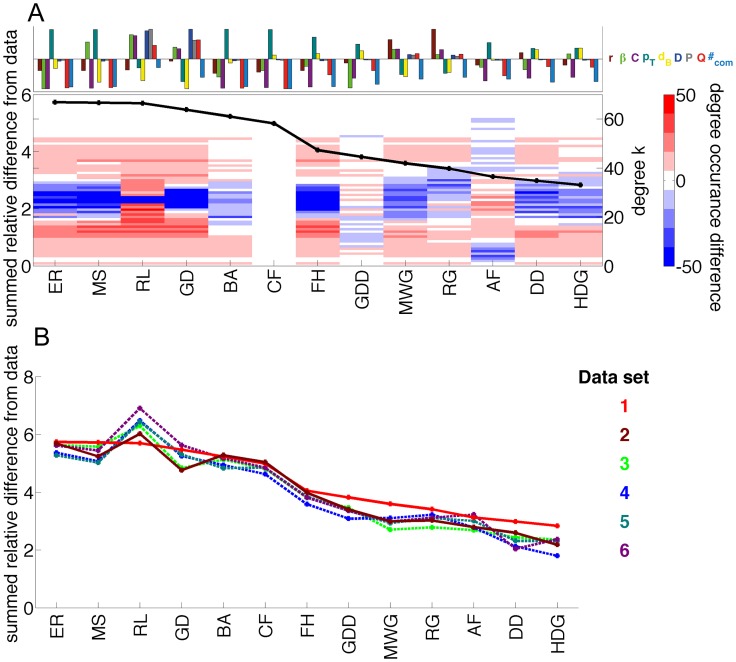
Comparison of the network models and brain data. *(A; Top Panel)* For each model, we illustrate how summary network statistics (Assortativity *r*, hierarchy *β*, clustering *C*, Rentian scaling 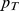, fractal dimension 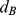, diameter *D*, mean path length *P*, modularity *Q*, and number of communities #*_com_*) differ from the same statistics extracted from empirical data. *(A; Main Panel)* The black line indicates the sum of the absolute values of the relative difference between each model and the data. The color image in the background indicates the difference between the degree distribution of the model and that of the data: red colors indicate that the model has too many nodes of a given degree, while blue colors indicate that the model has too few nodes of a given degree. Less saturated colors indicate more similarity between the degree distributions of the model and the data. *(B)* Colored lines indicate the sum of the absolute values of the relative difference between each model and the data from 6 separate diffusion imaging scans, acquired as described in [39].

Figure 11A provides a summary of graph diagnostics extracted from real and synthetic model data. We measure the relative difference between model and data, normalized by the value obtained from the model that fits the data the least for each diagnostic: 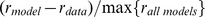. Models are placed in descending order, from those with the largest relative difference to the data (left-most side of the graph) to those with the smallest relative difference to the data (right-most side of the graph). We observe that embedded models generally have a smaller relative distance to the empirical data than non-embedded models. This result demonstrates that the brain is highly spatially organized, a fact that supports the view that physical constraints likely play an important role in large-scale properties of neurodevelopment.

### Integrated Insights

While the details of this set of analyses are of course important, we can also propose a set of integrated insights into the biological underpinnings of structural brain network organization based on the collective results extracted from these models. First, the fact that models hard-coded to display one topological property are unlikely to also display a second topological property suggests that the processes of neurodevelopment have not been selected by evolutionary drivers to optimize a single topological variable. Such a suggestion is intuitively plausible: What mechanisms exist to isolate and optimize single topological properties in the complicated cellular milieu of a developing organism? Evidence from evolution and development instead suggest that the neuronal systems in living organisms are constrained by energy and metabolic concerns [83]. While energetic concerns may subsequently translate into constraints on topological network architectures [12], [25], [28], topological features are unlikely to be the singular driving mechanism of evolution.

Supposing that energetic concerns play a role in guiding network connectivity in large-scale brain structure, how might these concerns manifest themselves in the observed network organization of a single organism at a single point in time? One possibility is that such constraints would impact on the physical length of connections since long connections are arguably more costly to both develop and maintain [12], [25], [28]. Consistent with this possibility, we observe that models that penalize physical length of connections (*embedded models*) tend to be more similar to the empirical data than models that hard-code specific topological properties (*non-embedded models*). This gross result, robust to individual variation in different model parameters, supports the view that biological physics may be a more fundamental driver of structural brain architecture than network topology.

However, we also note that simple distance models remain unable to capture all of the intricacies of the observed network architecture. While there is certainly room to construct more complicated physical models, it is also arguable that additional biophysical constraints are playing a secondary but influential role. A key feature of networked neuronal systems is their development over time, which displays complicated maturation-dependent trajectories [84]–[86]. It is therefore intuitively plausible that growth processes pose unique constraints on network development that cannot be captured by static physical distances alone. Indeed, we observe that 2 of the 3 models that display most similarity to the empirical network structure are growing models (the affinity model and the hybrid distance growth model, which we define for the first time in this paper), suggesting that principles underlying the time evolution of network structures is critical. If true, this result uncovers a major gap in current network models of neuronal systems: namely, a sequence of models of increasing complexity that account for both physical constraints and growth processes on final (adult) network architecture. We speculate that such models, which obey principles of both *physics* and *time*, will be best able to capture observed empirical brain network structure.

### Pragmatic Uses of Models and Model Batteries

Model interpretations aside, it is important to emphasize that this work has a complementary purpose: to provide researchers with mathematical null models to inform statistical inference. The pragmatic uses of these models fall under two broad categories: (i) the use of a single model and (ii) the use of the full model battery.

Single models can be used to address the question “How different are my two sets of networks in property *y* beyond that expected by their differences in property *x*.” For example, one might have a group of networks from a clinical population and a group of networks from a control population. The two groups might differ in both their degree distribution and their clustering coefficient. However, one would like to test whether their difference in clustering coefficient is more than expected given their difference in degree distribution. That is, one would like to isolate the independent contribution of one network parameter to the phenotype of the disease. The statistical test one could then employ is to compare the clustering coefficient of the empirical networks in one group (normalized by the clustering coefficient of the associated configuration models, which control for degree distribution) to the clustering coefficient of the empirical networks in the other group (again normalized by the clustering coefficient of the associated configuration models). Such a test directly determines whether the clustering coefficient is more different between the two groups than expected given the differences in their degree distributions. While we have used the clustering coefficient and degree distribution for simplicities sake in this argument, all other (potentially more complicated) pairs of properties can be examined similarly (e.g., hierarchical structure, preferential attachment, modular structure, wiring properties, etc.).

In addition to single models, model batteries can be used to probe more general questions of group differences between sets of networks, for example from clinical and control populations. In some group comparisons, it is possible to observe marginally significant group differences in many network properties but to not observe any single network property that is affected drastically in isolation. In such cases, it is useful to report a comprehensive statistical test that encompasses these findings, rather than report a series of separate *t*-tests. In this context, model batteries can be extremely useful because they can provide response functions (such as the summed relative difference from data, illustrated for a single individual in Figure 11A) that indicate the differences between the data and the model battery. Different individuals can have different response functions (as illustrated in Figure 11B), as can different groups. To directly compare these functions between groups, one can use a branch of statistics known as *functional data analysis* (for a relevant textbook see [87] and for an application in network neuroimaging see [88]). Model batteries therefore complement network diagnostics in providing measurable statistics that can be used to identify subtle differences in network architecture between groups.

In the following sections we discuss the details of each model more fully and relate our results to prior work. We conclude with a description of model interpretations, future directions, and methodological limitations.

### Non-embedded Models

We probe non-embedded models with differing amounts and types of structure. While the Erdös-Rényi model provides an important benchmark with a random topology, it bears little resemblance to the brain network. Although a homogeneous random distribution of links has been suggested to characterize the small-scale structure of neuron-to-neuron connections [89], [90], the large-scale structure of human and animal brains instead displays heterogeneous connectivity [67]. Perhaps one of the simplest measures of this heterogeneity is found in the degree distribution, which displays a predominance of low degree nodes and a long tail of high degree nodes. In comparing the degree distribution of the brain to that obtained from a BA model, it is clear that this tail, however, is not well-fit by a power-law, a finding consistent with previous reports in brain anatomy [21], [38] and function [15], [91]. However, by matching the empirical data, for example using a configuration model with the same degree distribution, we note that we do not automatically uncover higher order structures like assortativity, suggesting that the degree distribution provides only limited insight into the forces constraining brain network development.

Several decades ago, neuroanatomists observed that the *pattern* of connections in several animal brains displayed a combination of both densely clustered areas and long range projects between distant areas [92]–[95]. The regular lattice and Gaussian drop-off models are able to capture these densely connected structures but fail to capture the extent of long-range connectivity observed in the brain. The small-world modular and fractal hierarchical models contain both properties: dense local connectivity and long-range interactions. The fractal hierarchical model has the added benefit of containing nested structures, which have been implicated in the heterogeneity of neuronal ensemble activity [11] and in the separation and integration of information processing across multiple frequency bands [96]. Moreover, hierarchical modular structure has been identified in organization of white matter streamlines in human diffusion weighted imaging data [8], [72], [74] and implicated in neurobiological phenomena [11], [75], [76].

None of the non-embedded models discussed earlier in this section simultaneously provide a heterogeneous degree distribution, degree assortativity, hierarchical topology, and realistic topological dimensions. Such a “No Free Lunch” rule is perhaps unsurprising, in that a network that is developed to directly obtain one property typically fails to also display a second property. This result suggests that the topological properties that we explore here are in some sense independent from one another. It is, however, important to clarify that the interpretation of our findings in light of the observed correlations between network diagnostic values themselves, estimated over different networks or models (see previous literature, e.g., [97], [98], and results for the current data presented in Figure S4 in the Supplementary Materials), that suggest the need for methods to identify distinguishing properties among networks [56], [99]. The two sets of observations can be brought together by realizing that while classes of networks (e.g., brain networks) might display correlated network diagnostics values, these relationships need not be expected theoretically from any randomly chosen set of networks. Indeed, networks can be segregated into families based on the profile of interdependence between network diagnostic values [100].

Finally, in our affinity model, we hard-code both degree assortativity and a continuous hierarchical topology, rather than the discrete hierarchy employed in nested models like the fractal hierarchical model examined here. Interestingly, however, and in contrast to the other non-embedded models, we simultaneously obtain a heterogeneous degree distribution, and similar estimates of the topological dimension. This model fits multiple properties of brain networks that were not explicitly included in the construction of the network model, but are nevertheless a consequence of a three-parameter fit in the specific affinity model selected. The affinity model therefore serves as a promising candidate as both a generative model and statistical null model of brain organization.

### Embedded Models

In an effort to include additional biological constraints, we also explore several models that employ information regarding either the physical placement of network nodes or that place constraints on the Euclidean lengths of network edges. In general, this set of networks outperforms most of the non-embedded network models that we studied, demonstrating that the brain is highly spatially organized and supporting the notion that physical constraints might play important roles in brain network development and structure [8], [25]–[29], [90], [101], [102].

It is important to preface the discussion of our results by mentioning the fact that the properties of empirically derived brain networks display a heterogeneity that could at least in part stem from the peculiar physical properties of the organ. Brains are symmetric objects, with the two hemispheres being connected with one another via tracts in the corpus callosum and via subcortical structures. This separation allows for a very different topology *within* a hemisphere than *between* hemispheres. Moreover, cortical areas (gray matter) form a shell around the outer edges of the brain while their connections (white matter) compose the inner volume. Finally, brain areas are inherently heterogeneous in physical volume, making their distances from one another far from homogeneous. While the morphology of the brain constrains its potential topological properties, evidence also suggests that the lengths of tracts connecting brain areas follow a heavy tailed distribution, with short tracts being relatively common and long tracts being relatively rare [26], [27]. These findings are in concert with the idea that energy efficiency—to develop, maintain, and use neuronal wiring—remains a critical factor in brain evolution and development [29], [103].

In this study, we begin with a random geometric model, whose nodes are placed uniformly at random in a volume but whose edges selectively link nodes that are nearby in physical space. In light of the simplicity of this model, it is somewhat surprising that we obtain such good agreement with the empirical degree distribution, the presence of assortativity, and the presence of a hierarchical topology. In the minimally wired graph we employ a similar connection rule but also fix node placement to be identical to that in the empirical brain network, following previous studies [28]. However, neither of these two models are able to capture the extent of long-distance connections observed in the empirical data. By employing the distance drop-off model, we can fix a connection *probability* that varies with distance, rather than simply a connection *threshold*. This connection probability, however, is not enough to provide a realistically broad degree distribution. Our distance drop-off growth model combines the strengths of each of these models by laying down a set of seed edges uniformly at random in a volume and then iteratively adding edges between pairs of nodes according to a probability that falls off with inter-node distance. The resulting degree distribution and assortativity properties are the best match to the empirical data of the models that we studied. A hybrid between the minimally wired model and the distance drop-off growth model does not perform significantly better in matching these properties and shows a hierarchical structure that is more pronounced than the data.

Importantly, the embedded network models examined here are purposely simplistic. While arbitrarily more complex models could be constructed, our goal was to isolate individual drivers of topology and probe their relationship to observed network diagnostics. Other studies of interest in relation to these findings include those that explore the effects of geometric folding [90], radial surface architectures [102], and the effects of wiring minimization on functional networks [25].

### Model Interpretations

While the construction of network models is genuinely critical in providing null tests for statistical inference of brain structure from data, this avenue of research also has the potential to provide key insights into the neurobiological mechanisms of brain development and function if performed with appropriate caution. In light of this second use, we note that several of the network models discussed in this paper employ rules that are reminiscent of—or even directly inspired by—known biological phenomena. For example, physical models that place constraints on the length of connections in Euclidean space are consistent with the known distribution of connection lengths in the brain and the modern understanding of metabolic constraints on the development, maintenance, and use of long wires [26]–[29], [101], [103].

However, even topological constraints that link nodes that have similar sets of neighbors can be interpreted as favoring links between neurons or regions that share similar excitatory input [25]. As an example, our affinity model hard-codes two inter-node relationships. First, nodes with a similar degree are more likely to be connected to one another by an edge, leading to degree assortativity throughout the network. This behavior can be thought of as a mathematical representation of the intuitive principle of spatial homophily: large neurons with expansive projections (e.g., pyramidal or basket cells) are more likely to connect to one another because they densely innervate tissue over large distances. Network assortativity can also stem from the temporal homophily that occurs during development: neurons that migrate over longer distances during development are more likely to come into contact with—and therefore generate a synapse with—one another than neurons that migrate over shorter distances. The second topological relationship hard-coded into the affinity model is the prevalence of clustering in local neighborhoods, a property consistent with physical constraints on network development. As neurons develop, it is intuitively more likely for them to create synapses with neighboring neurons than non-neighboring neurons, thereby closing topological loops in close geographic proximity. While we have only provided a few examples here, links between topological rules and biological phenomena provide potentially critical neurophysiological context for the development and assessment of synthetic network models.

### Future Directions

The perspective that we have taken in choosing synthetic network models is one of parsimonious pragmatism. We seek to identify models with simplistic construction rules or growth mechanisms to isolate topological (non-embedded) and physical (embedded) drivers of network topology. One alternative perspective would be to begin with a certain graph topology (for example, an Erdős-Rényi graph), and iteratively rewire edges to maximize or minimize a network diagnostic or set of network diagnostics [25]. However, this approach requires prior hypotheses about which network diagnostics are most relevant for brain network development, a choice that is complicated by the observed correlations between such diagnostics [97]. Another approach is to employ exponential random graph models [16], [19], [104], which provide a means to generate ensembles of networks with a given set of network properties but do not provide a means to isolate mechanistic drivers of those network properties. A third approach is to construct a mechanistic model based on particle-particle collisions, which might serve as a physical analogy to the biological phenomena of neuronal migration through chemical gradients [105], [106]. In each of these cases, a perennial question remains: at what spatial scale should we construct these models to gain the most insight into the relevant biology? Important future directions could include the development of multiscale growth models, enabling us to bridge the scales between neuronal mechanisms and large-scale structure.

### Methodological Limitations

There remain important limitations to our work. In particular, we have focused on understanding the (binary) topology of brain network architecture rather than its weighted connection strengths. Our choice was informed by three factors: 1) An understanding of the relationship between synthetic network models and brain network topology could be useful for informing a similar investigation into network geometry, 2) In these particular networks, node degree (binary) and node strength (weighted by the number of streamlines) are strongly correlated (Pearson's correlation coefficient 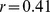, 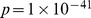) and therefore topology serves as a proxy for weighted connectivity, and 3) The choice of how to weight the edges in an anatomical network derived from diffusion imaging is an open one [107], and therefore investigations independent of these choices are particularly useful.

Network models constitute necessarily simplified representations of often very complex systems. The 13 synthetic network models we study in this work could be extended to include additional physical features of the human brain. For example, a key constraint on brain morphology and connectivity lies in the organ's bilateral symmetry. This symmetry in brain structure is evident in the distribution of anatomical connectivity in the brain networks examined in this study: pairs of homologous regions are more than 3 times more likely to be connected to one another than pairs of non-homologous regions. As described in [39], each of the 998 regions used in the parcellation is affiliated with one of 66 anatomical parcels defined based on surface reconstruction performed in Freesurfer. We calculated the average density of connections between all of the regions in one anatomical parcel and all of the regions in another anatomical parcel. In this way, we obtain a pairwise density of connectivity between all 66 anatomical parcels. The average density of connections between homologous regions is 15.22% and the average density of connections between non-homologous regions is 4.05%. The topological ramifications of this symmetry are not well understood.

Moreover, in simple network models, emphasis is placed on characterizing the patterns of network edges while the characteristics of individual nodes (apart from their connectivity) are examined to a lesser degree [108]. The development of more complicated models that account for feature vectors of brain region properties could provide additional insights into neurophysiological phenomena. Indeed, quantifying the relationship between a brain region's connectivity and its functional or anatomical properties is a critical goal of network neuroscience. Initial forays into this area have demonstrated that topological properties of a brain region (node degree) can be linked to neurophysiological properties (prevalence of amyloid-beta deposition) [109], suggesting the utility of network approaches in providing mechanistic hypotheses regarding disease attributes.

### Conclusion

In this paper, we have examined the mechanistic drivers of network topologies by employing and developing a range of synthetic network models governed by both topological (non-embedded) and physical (embedded) rules and comparing them to empirically derived brain networks. These tools may prove useful in the statistical inference of anatomical brain network structure from neuroimaging data. Future efforts can further build on these findings to identify neurobiologically relevant mechanisms for healthy brain architecture and its alteration in disease states.

## Supporting Information

Figure S1
**Empirical connection probability drop-off with physical distance.** The connection probability drop-off *g*(*r*) for *(A)* intra- and *(B)* inter-hemispheric connections. Empirical brain data is given by the data points: red indicates bins that were not utilized in the fits, blue indicates bins in which 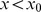, cyan indicates bins in which 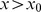, green indicates outlier bins excluded from fit. Fits are given by the lines: dotted line indicates the initial single truncated power-law fit, solid black line indicates the piecewise truncated power-law fit, and solid green indicates the piecewise truncated power-law fit with the interpolation to *g*(0) = 1.(EPS)

Figure S2
**Reliability of relational properties across data sets.** The *(i)* degree distribution (number *f* of nodes with a given degree *k_i_*), *(ii)* assortativity (correlation between a node's degree *k_i_* and the mean degree of that node's neighbors 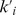, summarized by parameter *r*), and *(iii)* hierarchy (the relationship between the clustering coefficient *C_i_* and the degree *k_i_* over all nodes in the network, summarized by parameter *β*) for each of the six data sets separately shown in panels *(A)–(F)*. In panel *(A)*, data set 1 shown in grey was used in the visualizations provided in the main manuscript.(EPS)

Figure S3 **Reliability of the topological dimension estimates across data sets.** *(Main Panel)* The number of boxes as a function of the topological size of the box, estimated using the box-counting method [49] (see Materials and Methods) for the six empirical brain data sets. *(Inset)* The topological Rentian scaling relationship between the number of edges crossing the boundary of a topological box and the number of nodes inside of the box (see Materials and Methods) for the six empirical brain data sets.
(EPS)

Figure S4 **Correlation between network properties over empirical networks and models.** Each *ij^th^* element in this matrix represents the Pearson correlation coefficient between the values of network diagnostic *i* computed for all networks and models studied (Brain, ER, CF, RL, GD, MS, FH, BA, AF, RG, MW, DD, DDG, and HDG) and the values of network diagnostic *j* computed for the same networks and models. The color indicates the strength of the correlation with red colors indicating positive correlation and blue colors indicating negative correlation. In this matrix, we show the Pearson correlation coefficient between all possible pairs of the following network diagnostics: assortativity *r*, hierarchy *β*, clustering *C*, Rentian scaling *p_T_*, fractal dimension *d_B_*, diameter *D*, mean path length *P*, modularity *Q*, and the number of communities.
(EPS)

Table S1 **Parameter estimates for empirical connection density drop-off for the fits of** **Equation 2** **in Text S1 to intra- and inter-hemispheric data.**
(PDF)

Table S2 **Variance in network diagnostic values.** For each network or network model, we report the mean value of several network diagnostics as well as the estimated variance in those diagnostic values. Sources of variance that we report include the error (95*th* percentile) in the fit, the standard deviation of a diagnostic value estimated over 100 computations performed on the same network, and the standard deviation of a diagnostic value estimated over 100 realizations of a network model with the same parameter settings. The difference between the variance computed over 100 computations and that computed over 100 realizations is equal to the variance due to the model alone. For the original brain data and the minimally wired graph, we do not compute variance over realizations because these networks are deterministic. For the models in which the fits for the topological fractal dimension include only two data points, no fitting confidence interval is given.
(PDF)

Text S1 **Supplementary text.** In this Text S1 document, we include the following supporting materials: (i) a detailed description of parameter estimates for the distance drop-off models used (DD, DDG, HDG), and (ii) a description of correlations between network diagnostic values.
(PDF)
