# Supplementary material for: Resolving Structural Variability in Network Models and the Brain
Source: PLoS Comput Biol. 2014 Mar 27;10(3):e1003491. doi: 10.1371/journal.pcbi.1003491 (PMC3967917; doi:10.1371/journal.pcbi.1003491)
Supplement: Table S1 — Parameter estimates for empirical connection density drop-off for the fits of Equation 2 in Text S1 to intra- and inter-hemispheric data. (PDF) [file pcbi.1003491.s005.pdf]

Type	c	α	λ	x_0	γ
Intra-Hemisphere	0.1490	1.6608	0.2188	30.8774	0.8890
Inter-Hemisphere	0.0033	3.4492	0.2695	36.1939	4.0198

Table S1: Parameter Estimates for Empirical Connection Density Drop-Off for the fits of Equation 2 in Text S1 to intra- and inter-hemispheric data.
