# Supplementary material for: Resolving Structural Variability in Network Models and the Brain
Source: PLoS Comput Biol. 2014 Mar 27;10(3):e1003491. doi: 10.1371/journal.pcbi.1003491 (PMC3967917; doi:10.1371/journal.pcbi.1003491)
Supplement: Table S2 — Variance in network diagnostic values. For each network or network model, we report the mean value of several network diagnostics as well as the estimated variance in those diagnostic values. Sources of variance that we report include the error (95th percentile) in the fit, the standard deviation of a diagnostic value estimated over 100 computations performed on the same network, and the standard deviation of a diagnostic value estimated over 100 realizations of a network model with the same parameter settings. The difference between the variance computed over 100 computations and that computed over 100 realizations is equal to the variance due to the model alone. For the original brain data and the minimally wired graph, we do not compute variance over realizations because these networks are deterministic. For the models in which the fits for the topological fractal dimension include only two data points, no fitting confidence interval is given. (PDF) [file pcbi.1003491.s006.pdf]

	Brain	ER	CF	RL	GD	MS	FH	BA	AF	RG	MW	DD	DDG	HDG
**assortativity:** mean 100 iterations	0.1491	-0.001	-0.02	0.01	0.12	0.002	0.002	-0.035	0.07	0.53	0.3971	0.23	0.1	0.07
fitting confidence	0.0003	$5 \cdot 10^{-5}$	$7 \cdot 10^{-5}$	$4 \cdot 10^{-5}$	0.0001	$4 \cdot 10^{-5}$	$4 \cdot 10^{-5}$	$6 \cdot 10^{-5}$	$6 \cdot 10^{-5}$	0.0001	0.0002	0.0001	0.0002	$8 \cdot 10^{-5}$
standard deviation 100 computations	0	0	0	0	0	0	0	0	0	0	0	0	0	0
standard deviation 100 realizations	*NA*	0.009	0.01	0.01	0.01	0.009	0.008	0.010	0.03	0.03	*NA*	0.01	0.1	0.02
**hierarchy:** mean 100 iterations	0.2472	-0.11	0.11	0.54	0.39	0.45	0.16	0.03	0.15	0.30	0.3657	0.12	-0.10	0.31
fitting confidence	0	0.004	$6 \cdot 10^{-4}$	$2 \cdot 10^{-4}$	$3 \cdot 10^{-4}$	0.002	0.003	0.001	$5 \cdot 10^{-4}$	$1 \cdot 10^{-4}$	$2 \cdot 10^{-4}$	$1 \cdot 10^{-4}$	$7 \cdot 10^{-4}$	$7 \cdot 10^{-4}$
standard deviation 100 computations	0	0	0	0	0	0	0	0	0	0	0	0	0	0
standard deviation 100 realizations	*NA*	0.07	0.03	0.02	0.02	0.05	0.07	0.04	0.08	0.03	*NA*	0.04	0.09	0.04
**Rentian scaling:** mean 100 iterations	0.7446	0.953	0.952	0.683	0.587	0.952	0.898	0.954	0.86	0.644	0.635	0.819	0.849	0.821
fitting confidence	0.03	0.001	0.005	0.1	0.1	0.002	0.01	0.001	0.01	0.05	0.03	0.02	0.02	0.02
standard deviation 100 computations	0.003	0.003	0.005	0.001	0.002	0.002	$9 \cdot 10^{-4}$	0.002	0.004	0.002	0.003	0.004	0.004	0.003
standard deviation 100 realizations	*NA*	0.003	0.005	0.003	0.005	0.003	0.002	0.004	0.01	0.008	*NA*	0.004	0.006	0.004
**fractal dim.:** mean 100 iterations	3.7	3	4.0	2.1	1.5	2	4	3.4	3.6	2.7	2.4	4.4	4.3	4.5
fitting confidence	0.09	*NA*	*NA*	0.08	0.095	*NA*	*NA*	1.8	0.3	0.007	0.04	0.3	0.1	0.4
standard deviation 100 computations	0.2	0.4	0.1	0.03	0.05	0.3	0.2	0.1	0.2	0.04	0.07	0.2	0.2	0.3
standard deviation 100 realizations	*NA*	1	0.2	0.5	0.3	2	0.1	0.7	0.3	0.2	*NA*	0.3	0.5	0.6
**modularity:** mean 100 iterations	0.6151	0.1722	0.161	0.8223	0.8	0.176	0.373	0.169	0.36	0.69	0.7	0.50	0.44	0.49
standard deviation 100 computations	$3 \cdot 10^{-4}$	0.004	$3 \cdot 10^{-4}$	$9 \cdot 10^{-5}$	$2 \cdot 10^{-4}$	$5 \cdot 10^{-4}$	0	$3 \cdot 10^{-4}$	0	0	$2 \cdot 10^{-5}$	$6 \cdot 10^{-5}$	0	$3 \cdot 10^{-4}$
standard deviation 100 realizations	*NA*	0.003	0.003	$6 \cdot 10^{-4}$	0.005	0.001	0.004	0.002	0.04	0.01	*NA*	0.02	0.02	0.01
**# communities:** mean 100 iterations	18.46	3.9	4	14.0	9.0	4	4	4	7.7	9.3	8	6.4	5.3	6.7
standard deviation 100 computations	0.6	0.3	0	0.2	0.1	0	0	0	0	0	0	0	0	0.3
standard deviation 100 realizations	*NA*	0.14	0.1	1.0	0.14	0	0.1	0	4	1.4	*NA*	1.5	2.1	1.7

Table S2: Variance in Network Diagnostic Values. For each network or network model, we report the mean value of several network diagnostics as well as the estimated variance in those diagnostic values. Sources of variance that we report include the error ($95th$ percentile) in the fit, the standard deviation of a diagnostic value estimated over 100 computations performed on the same network, and the standard deviation of a diagnostic value estimated over 100 realizations of a network model with the same parameter settings. The difference between the variance computed over 100 computations and that computed over 100 realizations is equal to the variance due to the model alone. For the original brain data and the minimally wired graph, we do not compute variance over realizations because these networks are deterministic. For the models in which the fits for the topological fractal dimension include only two data points, no fitting confidence interval is given.
