# Supplementary material for: Resolving Structural Variability in Network Models and the Brain
Source: PLoS Comput Biol. 2014 Mar 27;10(3):e1003491. doi: 10.1371/journal.pcbi.1003491 (PMC3967917; doi:10.1371/journal.pcbi.1003491)
Supplement: Text S1 — Supplementary text. In this Text S1 document, we include the following supporting materials: (i) a detailed description of parameter estimates for the distance drop-off models used (DD, DDG, HDG), and (ii) a description of correlations between network diagnostic values. (PDF) [file pcbi.1003491.s007.pdf]

Supplemental Material for
Resolving structural variability in network models and the brain

Florian Klimm[1,2,3], Danielle S. Bassett[1,4,5,*], Jean M. Carlson[1], Peter J. Mucha[3,6]
[1]Department of Physics, University of California, Santa Barbara, CA 93106 USA
[2] Institut für Physik, Humboldt-Universität zu Berlin, 12489 Germany
[3] Department of Mathematics, University of North Carolina, Chapel Hill, NC 27599 USA
[4] Sage Center for the Study of the Mind, University of California, Santa Barbara, CA 93106 USA
[5] Department of Bioengineering, University of Pennsylvania, Philadelphia, PA 19104 USA
[6] Department of Applied Physical Sciences, University of North Carolina, Chapel Hill, NC 27599 USA
[*]Corresponding author email dsb@seas.upenn.edu

In this Text S1 document, we include the following supporting materials:

- Detailed description of parameter estimates for the distance drop-off models used (DD, DDG, HDG).
- Description of correlations between network diagnostic values.

Extracting Empirical Connection Probability Drop-off

In the distance drop-off (DD), distance drop-off growth (DDG), and hybrid distance growth (HDG) models we used a connection density g that depends on the Euclidean distance r between a pair of nodes. To tune our models, we estimated the empirical relationship between connection density and distance, hereafter referred to as *connection probability drop-off*, from the brain data. We observed that the probability drop-off of connections that lay within a single hemisphere displayed significantly different behavior than the probability drop-off of connections that lay between hemispheres. We did not observe systematic differences between the connection probability drop-off in the two hemispheres, and therefore treat all within-hemispheric connections identically without distinguishing connections that lay in the left hemisphere from connections that lay in the right hemisphere.

To estimate the connection probability drop-off function $g(r)$ from the empirical data, we used an adaptive binning algorithm that determines $\frac{\#\text{ connections}}{\#\text{ possible connections}}$ for each bin width Δ_r, where Δ_r is chosen to ensure that each bin contains on average 50 connections. To fit the data with sufficient precision, we first define a truncated power-law function using the form

$$f(x) = cx^{\alpha} \exp(-\lambda x) \tag{1}$$

where c is a constant, x is the minimum physical distance of connections in the bin, α is the power-law exponent, and λ is the natural exponent. However, we observed that this single truncated power-law fit was unable to adequately estimate the preponderance of long distance connections in the data. We therefore used a piecewise function with two truncated power-law functions of the form

$$g(x) = \begin{cases} f(x) & \text{if } x > x_0 \\ \frac{1}{2}f(x) + \frac{1}{2}f(x_0)\frac{x}{x_0}^{-\gamma} & \text{if } x < x_0 \end{cases} \tag{2}$$

where $f(x)$ is as defined in Equation 1, λ is the power-law exponent for all bins in which $x < x_0$, x_0 is the minimum physical distance at which the truncated power-law function begins to fit the data, and γ is the power-law exponent for all bins in which $x > x_0$. To minimize boundary and resolution effects, we excluded the bin with the smallest minimum physical distance and the bin with the largest minimum physical distance.

Algorithmically, we first fit a single truncated power-law function to the data to obtain estimates for the parameters c, α, and λ (see dashed line in Figure S1). We then used these estimates as initial parameters in the fit of Equation 2 to the data (see solid line in Figure S1). To obtain boundary values for the function, we performed a linear interpolation from the bin with the smallest minimum physical distance to the boundary value $g(0) = 1$ (see green line in Figure S1).

The results of this model, which contains 5 tunable parameters (c,α,λ,x_0,γ), are shown in Figure S1, where we observe a good agreement between the fit and the data. The estimated parameter values are provided in Table S1.

Description of Correlations Between Network Diagnostic Values

In Figure S4, we show the Pearson correlation coefficient between all possible pairs of network diagnostic values, estimated for the set of real and synthetic network models described in the main manuscript. We observe two separate families of network diagnostics whose values are strongly positively correlated over network models. The first family is composed of the Rentian scaling exponent p and the fractal dimension (estimated by box counting) d_B. The second family is composted of the hierarchy β, the number of communities, the modularity index Q, the network diameter D, the path length P, and the clustering coefficient C. The two families of network diagnostics are negatively correlated with one another. The assortativity r appears to be the most independent of the network diagnostics, displaying

values that do not correlate particularly well with either family, a finding that is consistent with prior observations in physical networks [1].

References

[1] Bassett DS, Owens ET, Daniels KE, Porter MA (2012) The influence of topology on sound propagation in granular force networks. Phys Rev E 86: 041306.
